# Genotyping-by-Sequencing and QTL Mapping of Biomass Yield in Two Switchgrass F_1_ Populations (Lowland x Coastal and Coastal x Upland)

**DOI:** 10.3389/fpls.2022.739133

**Published:** 2022-05-19

**Authors:** Rasyidah M. Razar, Peng Qi, Katrien M. Devos, Ali M. Missaoui

**Affiliations:** ^1^Institute of Plant Breeding, Genetics and Genomics, University of Georgia, Athens, GA, United States; ^2^Genetic Resources and Improvement Unit, RRIM Research Station, Malaysian Rubber Board, Selangor, Malaysia; ^3^Department of Crop and Soil Sciences, University of Georgia, Athens, GA, United States; ^4^Department of Plant Biology, University of Georgia, Athens, GA, United States

**Keywords:** QTL mapping, segregation ratio distortion, linkage maps, genotyping-by-sequencing, switchgrass, biomass

## Abstract

The prevalence of genetic diversity in switchgrass germplasm can be exploited to capture favorable alleles that increase its range of adaptation and biomass yield. The objectives of the study were to analyze the extent of polymorphism and patterns of segregation distortion in two F_1_ populations and use the linkage maps to locate QTL for biomass yield. We conducted genotyping-by-sequencing on two populations derived from crosses between the allotetraploid lowland genotype AP13 (a selection from “Alamo”) and coastal genotype B6 (a selection from PI 422001) with 285 progeny (AB population) and between B6 and the allotetraploid upland VS16 (a selection from “Summer”) with 227 progeny (BV population). As predictable from the Euclidean distance between the parents, a higher number of raw variants was discovered in the coastal × upland BV cross (6 M) compared to the lowland × coastal AB cross (2.5 M). The final number of mapped markers was 3,107 on the BV map and 2,410 on the AB map. More segregation distortion of alleles was seen in the AB population, with 75% distorted loci compared to 11% distorted loci in the BV population. The distortion in the AB population was seen across all chromosomes in both the AP13 and B6 maps and likely resulted from zygotic or post-zygotic selection for increased levels of heterozygosity. Our results suggest lower genetic compatibility between the lowland AP13 and the coastal B6 ecotype than between B6 and the upland ecotype VS16. Four biomass QTLs were mapped in the AB population (LG 2N, 6K, 6N, and 8N) and six QTLs in the BV population [LG 1N (2), 8N (2), 9K, and 9N]. The QTL, with the largest and most consistent effect across years, explaining between 8.4 and 11.5% of the variation, was identified on 6N in the AP13 map. The cumulative effect of all the QTLs explained a sizeable portion of the phenotypic variation in both AB and BV populations and the markers associated with them may potentially be used for the marker-assisted improvement of biomass yield. Since switchgrass improvement is based on increasing favorable allele frequencies through recurrent selection, the transmission bias within individuals and loci needs to be considered as this may affect the genetic gain if the favorable alleles are distorted.

## Background

Switchgrass, *Panicum virgatum* L., is a C4 perennial warm-season grass native to most of North America, spanning southern Canada, most of the United States, and northern Mexico (Casler, 2012). It has been used for pasture and rangeland grazing since the 1940s (Casler, 2012). It was selected by the U.S. Department of Energy (DOE) Biofuel Feedstock Development Program (BFDP) in 1991 (Kszos et al., 2000) as a herbaceous model species for biomass energy production due to its high biomass yield, low nutrient, and water requirement, and suitability for planting in marginal land unsuitable for grain and forage crops (Sanderson et al., 1996; Parrish and Fike, 2005).

Switchgrass has traditionally been divided into two major ecotypes, which are upland and lowland, based on their phenotypic divergence caused by the difference in latitudinal adaptation (Casler, 2012). Upland ecotypes are widely adapted to latitudes north of 34°N, extending into much of eastern Canada, while lowland ecotypes are adapted to the south, approximately up to 42°N in the western portion of the grassland range, but can be found as far north as 45°N in eastern North America (Casler, 2012). An adaptation of upland ecotypes involves phenology for a short-growing season and tolerance to cold winter temperatures (Lowry et al., 2014). On the other hand, lowland ecotypes are more adapted to a longer growing season and are less tolerant of cold temperatures. In terms of morphological characters, lowland ecotypes are taller, have fewer and larger tillers, longer and wider leaf blades, and thicker stems than the upland ecotypes (Casler, 2012). Recently, a third coastal ecotype with upland leaf type and lowland plant architecture, and occupying the same range as the lowland ecotype has been recognized (Lovell et al., 2021).

The basic chromosome number of switchgrass is × = 9, with a wide range of somatic chromosome counts, from *n* = 18 to 108 (Nielsen, 1944; Barnett and Carver, 1967). Lowland ecotypes are mostly tetraploid (2*n* = 4*x* = 36 chromosomes) and, rarely, octoploid (2n = 8x = 72) (Zhang et al., 2011a). Upland ecotypes can be tetraploid or octoploid, with the octoploids being approximately two to three times more abundant than the tetraploids. Zhang et al. (2011b) estimated the earliest divergence of upland and lowland ecotypes to be around 1.3 million years ago (MYA). Switchgrass is predominantly cross-pollinated due to gametophytic self-incompatibility (Martinez-Reyna and Vogel, 2002). Successful self-pollination has been reported, although selfing typically leads to inbreeding depression (Liu and Wu, 2012; Li et al., 2014; Adhikari et al., 2015; Dong et al., 2015). Crossing of upland and lowland ecotypes is possible at the same ploidy level and several studies have shown that the inheritance in tetraploid switchgrass is disomic with chromosome pairing occurring only between homologous chromosomes within a subgenome (Missaoui et al., 2005; Okada et al., 2010; Lu et al., 2013).

Switchgrass yield is largely determined by genotype, length of the growing season, the maturity of the stand, quality of the soil, and the availability of water and nutrients. At the most southern locations, lowland cultivars can be expected to produce at least twice the yield of upland cultivars; the yield, however, declines with increasing latitude. At the extreme northern locations, the biomass yield of lowland cultivars is reduced because of their inability to survive the cold winters (Casler et al., 2004). The biomass yield of lowland cultivars at the transition zone, where both ecotypes can be found, is 30–50% higher than that of upland cultivars (Casler, 2012). In the northern part of the US, typical switchgrass yields are 4.5–13.5 t/ha, in the middle part of the US, the yield ranges from 9 to 18 t/ha, while in the south, the yield is the highest, with 13.5 to 22.4 t/ha in drier areas and 33.6 t/ha or more in wetter and long growing season areas. As a warm-season grass that grows from late spring to early fall, switchgrass is typically harvested annually in fall. It may be possible to harvest switchgrass cultivars with an extended growth period twice, once in early summer and once in late fall.

Several genomic studies in switchgrass have contributed to the ample genomic resources currently available for the species. These include restriction fragment length polymorphism (RFLP) markers (Missaoui et al., 2005), simple sequence repeat markers (SSR) (Okada et al., 2010; Liu et al., 2012, 2013; Serba et al., 2013; Li et al., 2014), and more recently, GBS markers (Lu et al., 2013; Fiedler et al., 2015; Ali et al., 2019), sequenced cDNA libraries (Tobias et al., 2008; Wang et al., 2012; Palmer et al., 2015, 2017; Tornqvist et al., 2017), and exome capture array (Evans et al., 2014), sequenced bacterial artificial chromosome (BAC) libraries (Sharma et al., 2012), and a high-quality genome assembly (Lovell et al., 2021). Genetic and genomic studies in switchgrass have been greatly facilitated by the publicly available AP13 reference genomes. We originally used the *Panicum virgatum* V4.1 assembly, and then, transferred mapped markers to the most recent addition, *Panicum virgatum* V5.1 (https://phytozome-next.jgi.doe.gov/; Lovell et al., 2021). The V5.1 genome assembly totals 1,125.2 Mb, and consists of 1,090 ordered and oriented contigs (Contig N50 = 5.5 Mb).

This manuscript describes the construction of genetic maps in two switchgrass F_1_ populations derived from a cross between a winter dormant lowland genotype, AP13, and a non-dormant coastal genotype, B6 (AB population), and between B6 and a winter dormant upland genotype, VS16 (BV population). AP13 was chosen as a parent because of its high biomass yield when grown in southern locations. B6 is a non-dormant genotype that has a longer growing season in southern locations and can be harvested twice a year to enhance overall yield. VS16 is adapted to northern locations and can potentially contribute to cold tolerance in the population. The objective of crossing AP13 with B6 was to produce a population that segregates for high biomass yield and non-dormancy. On the other hand, B6 was crossed with VS16 to generate a population that segregates for non-dormancy and cold tolerance. These traits are crucial for extending the growing season to increase biomass yield while maintaining persistence in environments with mild winters.

Parents and the F_1_ progeny were genotyped using genotyping-by-sequencing (Elshire et al., 2011) and genetic maps were generated using a two-way pseudo-testcross mapping strategy, identifying recombination events that occurred at each parental side (during egg and pollen formation) (Grattapaglia and Sederoff, 1994; Daverdin et al., 2015). We used the SNP data from the three parents to calculate the Euclidean genetic distance to verify the level of diversity within each cross. We compared the polymorphism levels and the patterns of segregation distortion between the two crosses based on the SNP data generated. The study also seeks to examine whether a population derived from a wide cross such as between coastal × upland ecotypes experiences more segregation distortion of markers due to the existence of potential reproductive barriers between the divergent parents (Okada et al., 2010). Finally, we conducted a QTL mapping of biomass yield using the four linkage maps. Previously published inter-ecotypic QTL studies in switchgrass employed lowland × upland mapping populations. Our study is novel in that we employ populations generated from lowland × coastal and coastal × upland crosses.

## Materials and Methods

### Population Development and Collection of Biomass Data

Two F_1_ populations, derived from the crosses AP13 × B6 (AB population) and B6 × VS16 (BV population), were produced in the greenhouse in 2015 and 2016. B6 is a selection from PI 422001 that was collected in 1959 at Stuart, Martin County, Florida, and belongs to the ecotype group “coastal” (https://www.nrcs.usda.gov/Internet/FSE_PLANTMATERIALS/publications/flpmcrb5447.pdf. AP13 is a selection from “Alamo” switchgrass that was released in 1978 from the USDA Natural Resource Conservation Service (NRCS) in Knox City, Texas, and is a typical lowland type (https://www.nrcs.usda.gov/Internet/FSE_PLANTMATERIALS/publications/txpmcrb11189.pdf). VS16 is a selection from the upland accession “Summer,” which was originally collected from a prairie in Nebraska and released in 1964 by the South Dakota Agricultural Experiment Station (https://openprairie.sdstate.edu/cgi/viewcontent.cgi?article=1646&context=agexperimentsta_bulletins). AP13 and VS16 were the parents of the mapping population AP13 × VS16 (Missaoui et al., 2005).

The coastal genotype B6 stays green over the winter in Georgia and is considered non-dormant. Both AP13 and VS16 display winter dormancy. The three parents of the mapping populations are tetraploid (2*n* = 4*x* = 36). Both crosses were made by placing each set of parents close together in separate greenhouse sections to prevent unintentional cross-pollination from unidentified sources of switchgrass pollen. Seeds produced from cross-pollinated plants were harvested at maturity and were dried at room temperature before undergoing pre-chilling treatment to break seed dormancy. The pre-chilling treatment consisted of placing the seed on wet filter paper in a petri dish and putting the sealed petri dish in a 4°C refrigerator for 2 weeks. After that, the seeds were planted in flats for germination. Because of the limited amount of seed obtained for the BV population, the B6 × VS16 cross was repeated to generate additional progeny.

The seedlings from each population were genotyped with a single SSR marker that was described in Liu et al. (2013) (AB population; forward primer: AAGAGCAAACACATGCCAAG, reverse primer: GAAGTTCTGCTTAATGGCCC. BV population; forward primer: CTGCTTGCACACACCCAG, reverse primer: CTGGACAAGGGACGGTATCT) to ensure they were F_1_ hybrids before transferring them into bigger pots. The plants were later divided into three clonal replicates by separating the ramets. Three replicates of 285 AB progeny and the two AB parents, and three replicates of the first set of 66 BV progeny and both BV parents were transplanted in the field at the University of Georgia's Iron Horse Farm (IHF) in Greene County, GA (33.73° N,−83.30° W) in April 2017. Three replicates of the second set of 161 BV progeny were transplanted in the IHF in May 2018. The experimental design was a randomized complete block design with three replications. Plants were spaced by 3 feet (91.4 cm). The soil type is Cecil gravelly sandy loam. Field management included preemergence and postemergence (Pendimethalin and Atarazine) herbicide applications before planting and irrigation of the field after planting. Herbicide applications were repeated in the fall after harvest and in spring before the emergence of switchgrass.

The first-year harvest was done on September 24, 2018, using a Swift Machine forage plot harvester (Swift Machine and Welding Limited, Canada). The second-year harvest was done on September 29, 2019 using a Wintersteiger Cibus F/S harvester (Wintersteiger Seedmech, Austria). Plants were clipped at a height of 10 cm. The fresh biomass weight of the harvested material for each plant was measured immediately after clipping. For each plant, a subsample of 500 g was dried at 60°C in a convection oven for 2 days and then weighed for dry weight. The proportion of dry matter in the subsample was calculated in percentage, and estimation of whole-plant dry weight was done by multiplying the dry matter percentage with the whole-plant fresh biomass weight.

### Genotyping-by-Sequencing

Leaf tissue samples were collected from all the progeny and parents on ice and subsequently dried in a freeze drier (Labconco, Kansas City, MO, USA) for at least 2 days. Genomic DNA was extracted using the 2% hexadecyltrimethylammonium bromide (CTAB) method of Doyle and Doyle (1990). Genomic DNA quality was assessed by visualizing the samples on a 1% agarose gel. Genotyping-by-sequencing was done using the GBS protocol described by Qi et al. (2018), which was adapted from Poland et al. (2012). In brief, the genomic DNA of each parent and progeny was digested with two restriction enzymes, a “rare cutter” *Pst*I (New England Biolabs^®^, Ipswich, MA, USA, R0140S) and a “common cutter” *Msp*I (New England Biolabs^®^, Ipswich, MA, USA, R0106S). DNA fragments were then ligated to a barcoded adapter on the *Pst*I cut site and a common Y-adapter on the *Msp*I cut site. DNA fragments smaller than 300 bp were removed using Sera-Mag SpeedBeads^TM^ (Fisher Scientific^TM^, Thermo Fisher Scientific, Waltham, MA, USA, 09-981-123) at 1X volume. The size-selected DNA was PCR amplified for 16 cycles using Illumina forward and reverse primers. The DNA concentration of each sample was then measured using a Qubit^®^ 3 Fluorometer (Life Technologies, Thermo Fisher Scientific, Waltham, MA, USA) and sets of 150 samples were pooled in equal amounts. Two pooled sample sets per population were sent to the Georgia Genomics and Bioinformatics Core for fraction analysis, primer dimer filtration, and sequencing on a high-throughput flowcell (four lanes) on an Illumina NextSeq500 (paired-end 150 bp).

### Reads Filtering and Variant Calling

Following a quality check using FastQC, the reads were de-multiplexed according to their unique barcode sequences using “process_radtags” from the software Stacks (Catchen et al., 2011), and reads across the four lanes were merged into a single file for each genotype and type of reading (forward read 1 and reverse read 2). Reads were then trimmed to remove enzyme cut sites and reads with low-quality base (Phred score of <33) were discarded using FASTX_Toolkit version 0.0.14. The switchgrass reference genome version 4.1 downloaded from Phytozome v12.1.6 (https://phytozome.jgi.doe.gov/pz/portal.html) was used to align the trimmed reads using Bowtie2 version 2.2.9 (Langmead et al., 2009). Reads for which both the forward and reverse reads aligned to the genome sequence were processed for the Genome Analysis Toolkit (GATK) (McKenna et al., 2010) compatible format using Samtools version 1.3.1 (Li et al., 2009) and Picard version 2.4.1 (Broad Institute, 2018). SNP and indel calling was done on each genotype's output file using “HaplotypeCaller” from GATK version 3.4.0 (McKenna et al., 2010) and results were pooled across genotypes using GATK's “GenotypeGVCFs.” As we did not differentiate between SNPs and indels, we use the term SNP to refer to both marker types throughout the manuscript.

The SNP output file generated by “GenotypeGVCFs” from GATK 3.4.0 was filtered using VCFtools (Danecek et al., 2011) using the following criteria: (1) Quality score of >20 *(-min 20*); (2) Genotype quality score of >20 *(-minGQ 20*); (3) <20% of missing values (–*max-missing 0.8*); (4) Biallelic (–*min-alleles 2*; –*max-alleles 2*); (5) Minor allele frequency >5% (–*maf 0.05*); and (6) Read depth in single genotype at SNP position of ≥8 (–*minDP 8*). We also removed adjacent SNPs with an in-house Perl script as these SNPs had a higher likelihood of resulting from reading misalignments, and loci that were not detected in either parent using GATK's VariantToTable algorithm. The remaining filtered variants in the VCF file were then converted to a binary format using PLINK 1.07 (Purcell et al., 2007). Genotypes were recoded as “0” for homozygous reference alleles, “1” for heterozygous alleles, “2” for the alternative homozygous allele, and NA for missing data. To confirm the hybrid status of each progeny, markers that are homozygous for different alleles in the two parents (4,195 markers for AB and 5,186 markers for BV) were used as these should yield F_1_ progenies that were heterozygous at these loci. Progeny were considered true hybrids if they were heterozygous in at least 80% of all the loci analyzed. Progeny with ≤ 80% of heterozygous loci and progeny with >50% of missing data were removed.

### Construction of Linkage Maps

Variants with single-dose alleles (SDA; heterozygous in one parent and homozygous in the other parent) and a chi-square *p*-value for deviation of a 1:1 ratio >1 × 10^−15^ were selected for map construction. Based on our experience, markers with a chi-square *p*-value ≤ 1 × 10^−15^ are typically low-quality markers. Linkage maps were generated using JoinMap 5.0 (Van Ooijen, 2011) with the following settings: (1) Outcrosser population type (CP); (2) Independence LOD ≥ 7 for marker grouping; (3) Regression algorithm; (4) Kosambi function; (5) Recombination frequency <0.40; and (6) Jump threshold of 5 for removal of loci.

For a pseudo-testcross design, BC1 or DH are used as population types to correctly calculate the genetic distance. In our study, the JoinMap analysis represented an intermediate step in the mapping process, and the CP output provided information on the linkage phase of the markers, which we used to adjust marker scores to the correct format for MAPMAKER (see below).

The first step in linkage map construction was to exclude cosegregating markers. Then, the non-distorted SDA alleles with *p* > 0.05 were used for initial marker grouping to form a framework map (Grattapaglia and Sederoff, 1994; Fiedler et al., 2015). The next step was to add the rest of the markers (1 × 10^−15^<*p* < 0.05) to their strongest cross-link (SCL) loci in the framework map using the LOD threshold value of 10. This step is important to maintain the integrity of linkage groups (LGs) by giving the strongest link LGs in the initial step and to maximize the number of LGs (Fiedler et al., 2015). For tetraploid switchgrass, the expected number of LGs is 18, representing the nine “K” and nine “N” subgenome chromosomes of switchgrass. LGs were labeled according to the pseudomolecule name in the switchgrass reference genome 4.1 with each LG having at least 85% of the markers belonging to the same pseudomolecule.

The second round of mapping was carried out using a modified version of MAPMAKER (Qi et al., 2018), which, in our experience, is less user-friendly but yields more robust maps (Serba et al., 2013). Using the JoinMap maps as a starting point, we removed progeny and markers with >20% missing data. We had earlier used VCFtools to remove SNPs with >20% of missing data, but this filtering step had been completed before progeny classified as selves and progeny with >50% of missing data had been removed. Progeny removal increased the proportion of missing data in some SNPs to above the 20% threshold. We also removed markers that deviated from a 1:1 segregation at a *p*-value of <10^−10^ as many carried hallmarks of being low-quality. Finally, we removed end-markers that were distal by >10 cM, for which either the physical location or the segregation distortion indicated that these markers were low quality and had been artificially pushed to the end of the chromosomes during the mapping process. Since MAPMAKER is designed for inbred species with a known linkage phase, the phase of markers in an outcrossing species needs to be adjusted to correctly define recombination intervals. For this step, the genotypic scores for selected marker loci were reversed according to the linkage phase information ({1-} or {0-}) given by the JoinMap data output so that all marker genotypes were standardized to one linkage phase (Supplementary Figure 1). The markers in each linkage group were then ordered using MAPMAKER's “order,” “try,” and “ripple” commands. Population type was set to BC1 and recombination fractions were converted to genetic distances using the Kosambi function. Double recombination events were ignored using the “error detection on” function. Linkage maps were drawn using MapChart 2.30 (Voorrips, 2002).

### Analysis of Segregation Distortion

For this analysis, only mapped markers with <10% of missing data were considered. Log2 values of the A/H allele ratio were plotted, along with the nine K and N subgenome linkage maps. A/H is the ratio of A to H alleles in the population, which should be 1 for single-dose dose markers that segregate in a Mendelian fashion (1:1). Mapping intervals consisting of at least seven contiguous markers with significant distortion were indicated with ^*^, ^**^, ^***^, or ^****^ signifying that the highest level of distortion was significant at the 5%, 1%, 0.1%, or 0.01% level, respectively. The criterion of seven neighboring markers was chosen so that no undue weight would be given to outliers caused by non-random missing data or low-quality scores. Distorted regions on the same chromosome that were separated by at least seven markers with Mendelian ratios or for which the direction of the distortion was different were annotated separately.

### Estimation of Genetic Distance Between Parents

The SNPs with genotype calls in all three parents were used to calculate the Euclidean genetic distance between the parents to estimate their degree of genetic dissimilarity and make inferences on their genetic divergence. The similarity between two parents was first calculated using the formula:


$$\begin{array}{l} \frac{Number\;of\;identical\;loci}{Total\;number\;of\;loci\;} \end{array}$$

The Euclidean genetic distance between two parents was then calculated using the formula:


$$\begin{array}{l} \sqrt{1-Similarity} \end{array}$$

(Gower and Legendre, 1986).

### Colinearity of the Linkage Maps With Switchgrass Reference Genome V5.1

Variant-calling was initially done using switchgrass reference genome V4.1 for reading alignment. To determine the level of colinearity between the genetic map and the switchgrass reference genome assembly V5.1 (https://phytozome-next.jgi.doe.gov/), we conducted a BLASTN search using 50 bp of upstream sequence and 50 bp of the downstream sequence of each mapped SNP against switchgrass assembly V5.1 using an *E*-value <1 × 10^−5^ as matching hit threshold. The top hit was selected as the corresponding physical position.

### Statistical Analyses

A mixed model analysis of variance was conducted using the PROC MIXED in SAS 9.4 (SAS Institute Inc., Cary, NC, USA) to test the effect of genotype, year, and genotype by year (GxY) interaction on biomass yield. Genotype was treated as a fixed effect and year and GxY as random effects. The least-square (LS) means across replicates in each year were calculated using the LSMEANS statement in PROC MIXED. In addition, best linear unbiased predictors (BLUPs) were also calculated to transform the biomass value across replications, years, and population sets. To generate BLUP values, year and replications within a year were treated as fixed effects for AB, while year, replications within a year, and planting date were treated as fixed effects for BV. Genotype was treated as a random effect for both populations.

### QTL Mapping

The LS means of biomass weight for each year's harvest (2018 and 2019) and BLUP values (across reps, years, and populations) were used for QTL mapping (Supplementary File 1). LS means were used for QTL mapping for each year as this represents the mean value of the biomass weight (in its true measure) for the respective year. On the other hand, BLUP is a better method to transform data that consist of several replications, years, and population sets. The advantages of using BLUP include shrinkage of values toward the mean and maximizing the correlation between true and predicted genotypic values (Piepho et al., 2008). Linkage maps for each parent and marker profiles of the progeny were used together with the biomass weight values to conduct QTL mapping using Composite Interval Mapping (CIM) in R/QTL (Broman et al., 2003), which was run in R version 4.0.1 (R Core Team, 2021). The settings for CIM were a window size of 10 and the number of marker covariates (*n.marcovar*) used was 4. The genome-wide significance threshold for QTL was set using a permutation test (α = 0.05, *n* = 1,000) for each trait.

## Results

### Genotyping-by-Sequencing

#### AB Population (AP13 × B6)

The total number of reads generated for the whole population was 2,200,716,698 (2.2 B). Following the removal of low-quality reads, the number of reads per sample ranged from 72,292 to 16,657,730, with an average of 6,204,226 (6.2 M) and a size range of 20−142 bases. The mean GC content was 50.7% and the mean Phred score per reading was 33.9.

#### BV Population (B6 × VS16)

The total number of reads from the sequencing output was 2,372,553,906 (2.4 B). The number of high-quality reads per sample ranged from 467,566 to 33,662,486 with an average of 7,187,731 (7.2 M) reads with a size range of 21−142 bases. The mean GC content was 48.6% and the mean Phred score per reading was 35.

### Variant Calling

#### AB Population (AP13 × B6)

The mean percentage of reads aligned to the reference genome V4.1 was 65.1% (3,984,908 aligned reads/sample). The total number of variants in the raw VCF output after the GATK HaplotypeCaller and GenotypeGVCFs processes was 2,539,025 (2.5 M). Removal of loci and individual genotypes with a quality score <20, loci with >20% of missing data, non-biallelic loci, loci in a single genotype with a read depth <8X, and loci with a minor allele frequency (MAF) <0.05 resulted in 19,830 variants. Removal of variants that were missing in either parent reduced the number of SNPs to 14,816.

Hybrid individuals were identified using markers that were homozygous and fixed for different alleles in each parent. One progeny was heterozygous at 0.15% of the assessed loci and, hence, considered a self, and was discarded. An additional 12 progeny were discarded because of >50% missing data, leaving a final 285 progeny for the first stage of linkage-mapping. Following extraction of SDA markers and filtering for the goodness of fit to a 1:1 allele segregation ratio (chi-square *p*-value > 10^−15^), a total of 3,548 (24% of filtered variants) and 3,823 (26% of filtered variants) SNP markers were obtained that segregated in the maternal (AP13) and paternal parent (B6), respectively. A total of 398 SNP markers (2.7% of filtered variants) were heterozygous in both parents (single dose alleles present in both parents).

#### BV Population (B6 × VS16)

The mean alignment rate of reads to the reference genome was 73.3% (5,253,577 reads/sample). The raw SNP number identified was 6,043,505 (6 M), which was reduced to 31,374 after the filtering steps. A total of 27,517 variants remained when variants that were missing in either parent were discarded. Sixty-six progeny were discarded based on their genotypic data (heterozygous loci ranging from 34.30 to 64.33%), and an additional five progeny were discarded because of >50% missing data, leaving 227 progeny for the first phase of linkage mapping. After selection of SDA markers based on their goodness of fit to a 1:1 allele segregation ratio (chi-square *p*-value > 10^−15^), 5,693 (21% of filtered variants) and 7,883 (29% of filtered variants) markers were obtained that segregated in the maternal (B6) and paternal parent (VS16), respectively. A total of 758 SNP markers (2.8% of filtered variants) were heterozygous in both parents.

### Linkage Maps

#### AB Population (AP13 × B6)

**Figure 1:**
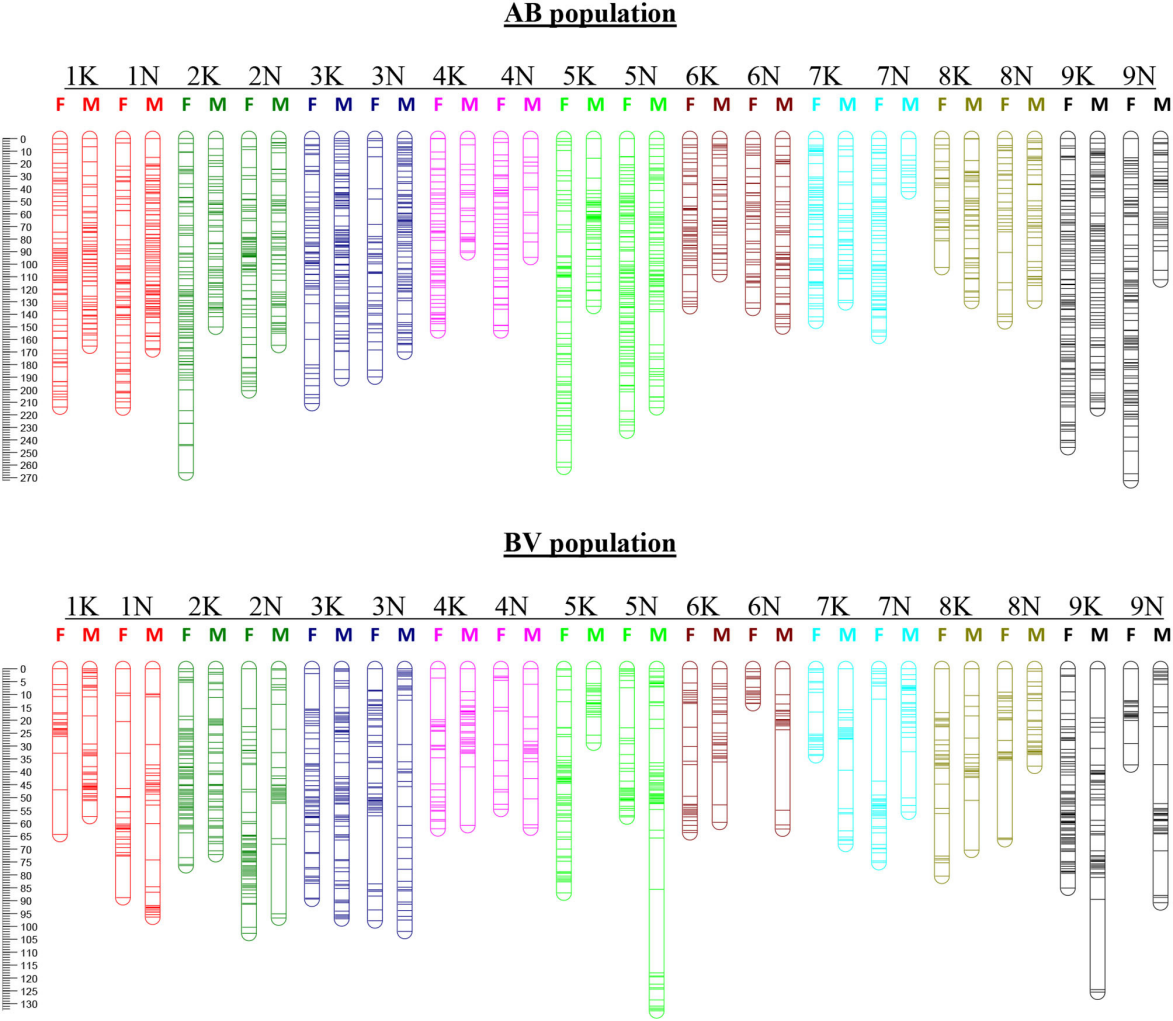
Linkage and homology groups of maternal (F) and paternal (M) maps for the AB population (top) and BV population (bottom). Genetic distance (in Kosambi centiMorgans) is given on the left-hand side. Individual bars represent loci positions with thicker bars indicating two or more closely linked loci. The LGs (numbered 1K-9K and 1N-9N) are specified above the corresponding female and male chromosomes.

Using JoinMap, 45 markers that cosegregated with another marker were discarded from the maternal data set. Eight hundred seventy-one Mendelian markers (chi-square *p*-value > 0.05) were used in the construction of a framework map. The remaining markers with distorted segregation ratios (0.05 > chi-square *p*-value > 1x10^−15^) were added to the framework LG, with which they had the strongest linkage. A total of 1,540 markers (43% of the total maternal SDA markers) could be ordered within the LGs. From the JoinMap output, we removed progeny and markers with >20% missing data, low-quality end markers, and markers deviating from a 1:1 segregation ratio with a chi-square *p*-value <10^−10^, and conducted a second round of marker ordering using MAPMAKER. The MAPMAKER analysis that led to the generation of the final AP13 maps was done on a total of 263 progeny and yielded a genetic map of 1,332 markers with a total genetic length of 3,435 cM (Table 1; Figure 1; Supplementary File 2—“AP13 map_AB population” tab).

**Table 1:** Marker numbers, map length (cM), and average inter-marker distances for each parental map in AP13 × B6 and B6 × VS16 populations.

**LG**	**AP13 × B6**						**LG**	**B6 × VS16**					
	**AP13**			**B6**				**B6**			**VS16**		
	**No. of markers**	**Map length (cM)**	**Average inter-marker distance (cM)**	**No. of markers**	**Map length (cM)**	**Average inter-marker distance (cM)**		**No. of markers**	**Map length (cM)**	**Average inter-marker distance (cM)**	**No. of markers**	**Map length (cM)**	**Average inter-marker distance (cM)**
1K	87	213.8	2.5	68	165.3	2.5	1K	54	64.3	1.2	134	57.3	0.4
1N	77	214.5	2.8	106	168.2	1.6	1N	63	88.8	1.4	84	96.4	1.2
2K	101	266.2	2.7	70	150.1	2.2	2K	139	76.4	0.4	116	72.1	0.6
2N	71	200.7	2.9	68	164.7	2.5	2N	181	102.7	0.6	81	96.7	1.2
3K	69	211.0	3.1	102	191.0	1.9	3K	132	89.4	0.7	150	97.0	0.7
3N	39	189.8	5.0	102	170.0	1.7	3N	115	97.8	0.9	63	101.9	1.6
4K	65	153.0	2.4	22	90.7	4.3	4K	42	62.1	1.5	97	60.8	0.6
4N	52	153.0	3.0	16	94.6	6.3	4N	30	54.4	1.9	55	61.8	1.1
5K	90	261.7	2.9	64	133.6	2.1	5K	123	87.0	0.7	62	28.8	0.5
5N	110	232.8	2.1	86	214.3	2.5	5N	53	57.5	1.1	153	132.7	0.9
6K	63	133.8	2.2	40	108.3	2.8	6K	51	63.6	1.3	74	59.6	0.8
6N	51	135.2	2.7	52	149.9	2.9	6N	39	13.5	0.4	42	62.2	1.5
7K	75	145.4	2.0	35	130.7	3.8	7K	25	33.7	1.4	83	68.1	0.8
7N	83	157.4	1.9	14	42.2	3.2	7N	102	75.2	0.7	60	55.6	0.9
8K	31	102.6	3.4	52	129.6	2.5	8K	49	80.5	1.7	54	70.4	1.3
8N	35	145.8	4.3	47	129.4	2.8	8N	54	66.3	1.3	70	37.8	0.5
9K	118	246.1	2.1	94	215.1	2.3	9K	158	85.1	0.5	125	125.5	1.0
9N	115	272.4	2.4	40	112.4	2.9	9N	45	37.3	0.8	95	90.8	1.0
	**1,332**	**3,435.2**	2.8*	**1,078**	**2,560.1**	2.8*		**1,509**	**1,235.7**	1.0*	**1,598**	**1,375.5**	0.9*

LG, linkage group. Values in bold are sum values except for values at asterisk (*) which are mean values.

For markers segregating in the paternal parent, 65 co-segregating markers were discarded and a total of 1,171 Mendelian markers were used in the construction of the framework map. An additional 61 distorted markers were added to the framework map, yielding a JoinMap PhaseI map with 1,232 markers (32% of the total paternal SDA markers). The PhaseII mapping, using MAPMAKER, was conducted on 267 progeny and yielded a B6 genetic map of 1,078 markers with a genetic length of 2,560 cM (Table 1; Figure 1; Supplementary File 2—“B6 map_AB population” tab).

The extent of recombination (cM/Mb) was significantly higher in the maternal AP13 parent than in the paternal B6 parent (paired *t*-test; *p* = 0.0013). Only the pairs of chromosomes that varied by <10% in the physical length they are covered were included in this comparison (Supplementary File 3).

Both the AP13 and B6 maps showed a high level of colinearity with the physical position of the mapped markers in the switchgrass reference genome V5.1, though there was some local misordering in low recombination regions, which likely correspond to the centromeric regions (Supplementary Figures 2a,b). The 100 bp of sequence surrounding each mapped SNP marker that was used to project the linkage maps onto assembly V5.1 is provided in Supplementary File 4. Combining the AP13 and B6 maps based on the physical location of the markers showed large regions that were only represented in either the AP13 map or the B6 map, suggesting the presence of largely homozygous tracts of significant length in both parents (Supplementary File 5—“AP13_B6_AB_KN” tab).

#### BV Population (B6 × VS16)

For the construction of the maternal map, 547 cosegregating markers were discarded. The PhaseI B6 maps consisted of 1,824 markers (47% of the total maternal SDA). The PhaseII B6 maps, constructed using 213 progeny, consisted of 1,509 markers and spanned 1,236 cM (Table 1; Figure 1; Supplementary File 2—“B6 map_BV population” tab). Of the paternal markers, 904 cosegregating markers were discarded and a total of 1,942 markers (44% of the total paternal SDA) were ordered within LGs of the PhaseI VS16 map. PhaseII mapping was conducted on 214 progeny. The resulting VS16 maps consisted of 1,598 markers and had a total genetic length of 1,376 cM (Table 1; Figure 1; Supplementary File 2—“VS16 map_BV population” tab).

Recombination was not significantly different between the maternal B6 parent and the paternal VS16 parent (Supplementary File 3). Similar to the AB population, high levels of colinearity were observed in the BV population between the physical and genetic maps (Supplementary Figures 2c,d). The BV maps lend further support to the presence of regions of 10 Mb or more in length that is predominantly homozygous in the B6 parent. Similar stretches were also identified in the VS16 parent (Supplementary File 5—“B6_VS16_BV_KN” tab).

### Segregation Ratio Distortion

#### AB Population (AP13 × B6)

**Figure 2:**
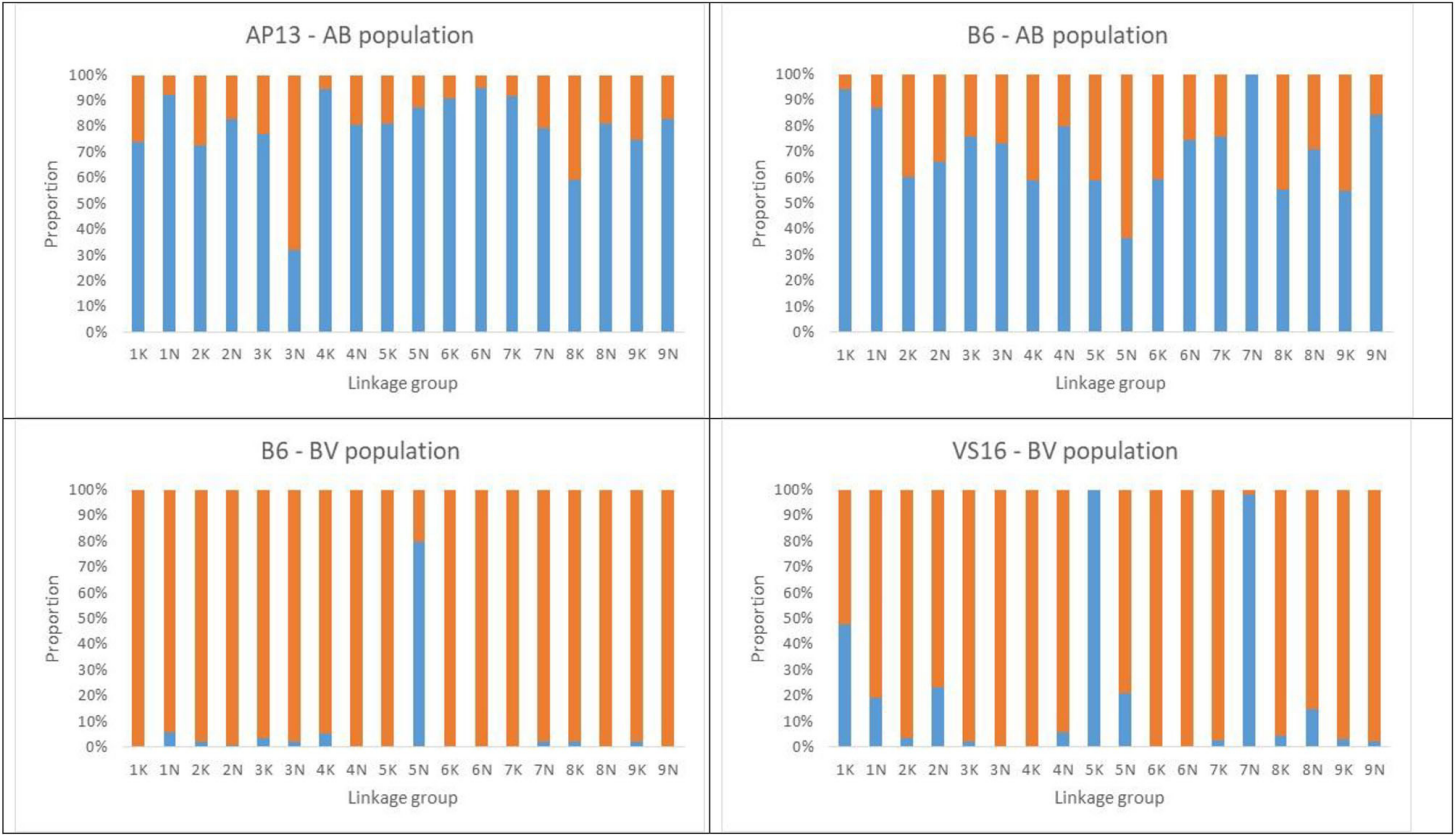
Proportion of distorted (blue bars) and Mendelian markers (orange bars) across LGs assessed by the Chi-square (*X*^2^) values for the goodness-of-fit test to 1:1 expected segregation ratios (*p* < 0.05).

The percentage of markers (<10% missing data) with distorted segregation ratios (chi-square *p*-value <0.05) in the population was 75% and ranged from 32 to 95% per LG in the maternal map and from 37 to 100% per LG in the paternal map. The percentage of distorted markers was higher than 50% in all LGs, except for LG 3N on the maternal map, and LG 5N on the paternal map (Figure 2). The highest frequency of distorted allele ratios was observed in LG 6N (95%) on the maternal map and LG 7N (100%) on the paternal map (Supplementary File 6). A total of 27 regions that contained at least seven consecutive distorted markers and were separated from another distorted region on the same linkage group by at least seven consecutive markers with Mendelian segregation ratios were identified on 17 LGs in the AP13 map (AB population) (Supplementary Figure 3A). Similarly, 23 distorted regions were identified on 17 LGs in the B6 map of the AB population (Supplementary Figure 3B).

#### BV Population (B6 × VS16)

In contrast to the AB population, more than 80% of markers in the BV population were segregated in a Mendelian fashion. Exceptions were LG 5N in the maternal map and LGs 5K and 7N in the paternal map, in which more than 50% of the markers deviated from a 1:1 segregation ratio (Figure 2; Supplementary File 6). The percentage of distorted alleles across LGs ranged from 0 to 80% in the maternal map and 0–100% on the paternal map. Using our definition of a distorted region (consisting of seven consecutive distorted markers), only one such region was identified in the B6 map, and four regions on four LGs in the VS16 map of the BV population (Supplementary Figures 3c,d).

### Estimation of Genetic Distance

To determine the Euclidean genetic distance between the parents, SNP-calling for the three parents was done separately to recover a higher number of SNPs. A total of 34,011 SNPs present in all three parents were used for the calculation of genetic similarity and Euclidean distance (Table 2). Parents with the highest genetic similarity are AP13 and VS16, with the fraction of shared loci exceeding half of the total SNPs. Parents with the highest genetic divergence are B6 and VS16.

**Table 2:** Genetic similarity and Euclidean distances calculated between parents.

	**AP13 and B6**	**B6 and VS16**	**AP13 and VS16**
Genetic similarity	0.225	0.159	0.545
Euclidean distance	0.880	0.917	0.674

### Phenotypic Trait Distribution

**Figure 3:**
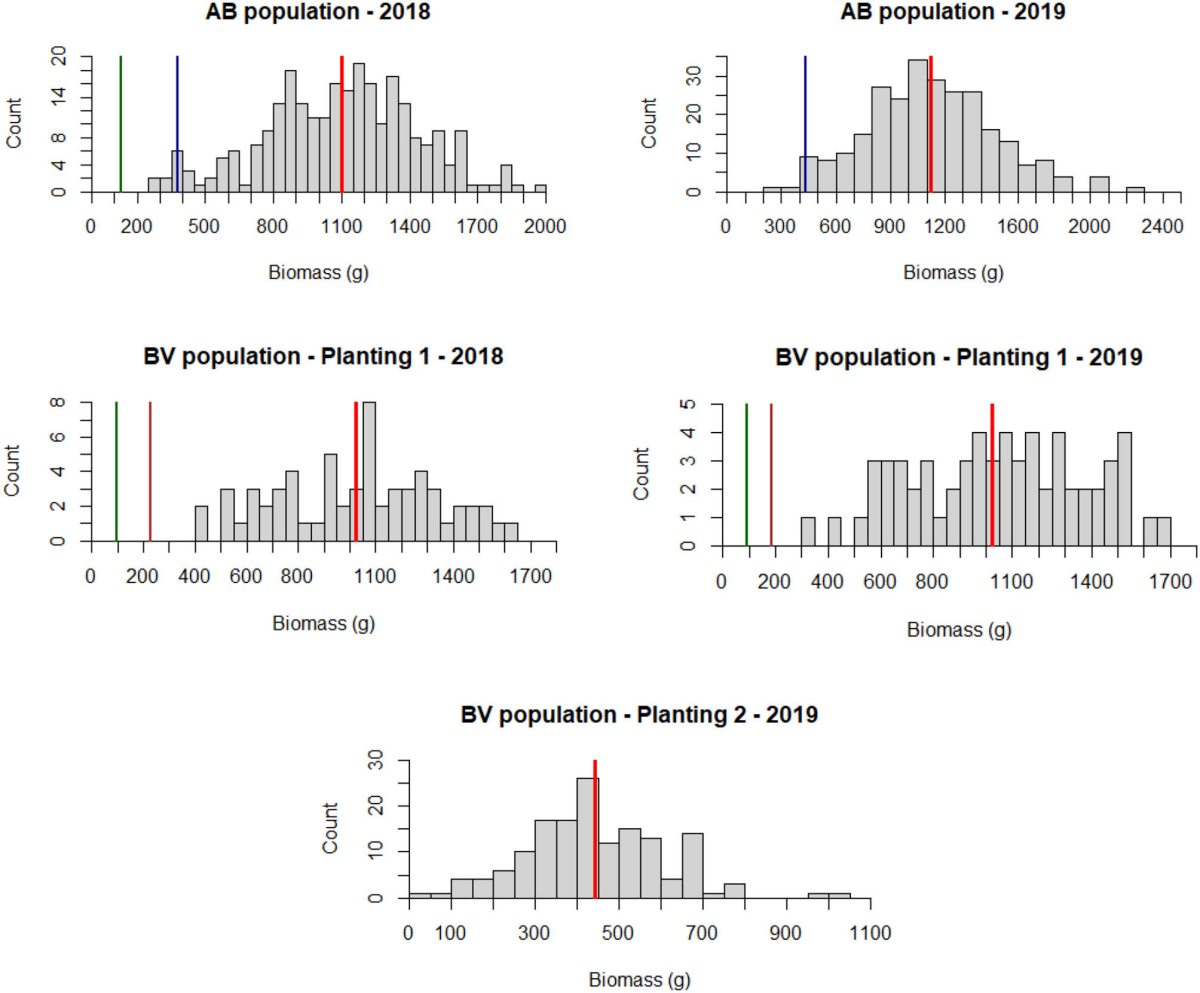
Phenotypic trait distribution of dry biomass weight in grams for AB and BV populations for 2 years of field evaluation (2018 and 2019). The first set of the BV population was phenotyped for both years while the second set was phenotyped in 2019. Redline = population mean; Greenline = B6 parent, Blueline AP13 parent; Brown line = VS16 parent. B6 plant from the AB population was dead during the second year of planting. For the BV population, parent plants were only planted in the plot containing the first set of the population.

The AB population had a higher dry biomass yield than the BV population in both years of evaluation (Figure 3). Dry biomass yield per plant in the AB population ranged from 263 to 2,094 g (mean = 1,102 g) in 2018 and 268–2,223 g (mean = 1,125 g) in 2019. For BV planting date 1, the ranges were 403–1,643 g plant^−1^ (mean = 1,023 g) in 2018 and 303–1,673 g plant^−1^ (mean = 1,047 g) in 2019. For BV planting date 2, the range was 25–1,006 g plant^−1^ (mean = 442 g) in 2019. Analysis of variance showed a significant genotype effect and non-significant year and GxY interaction effects in both populations (Table 3).

**Table 3:** Analysis of variance of biomass weight in both populations evaluated in 2018 and 2019.

**Source of variation**	**AP13 × B6**		**B6 × VS16 (P1)^A^**		**B6 × VS16 (P1)^B^**	
	**df**	**Mean square**	**df**	**Mean square**	**df**	**Mean square**
Genotype	284	598,991**	65	453,828**	159	76354**
Year	1	169,064^ns^	1	9,036^ns^	–	–
Genotype × Year	284	81,690^ns^	64	78,151^ns^	–	–
Rep (year)	4	1,019,631**	4	424,113*	2	11,197^ns^
Residuals	1,109	202,028	237	164,311	299	36,280

A *BV set 1 that was planted in April 2017;*

B *BV set 2 that was planted in May 2018,*

** *p < 0.01;*

* *p < 0.05.*

ns *not significant at p < 0.05*.

### Significant QTL for Biomass Yield

**Figure 4:**
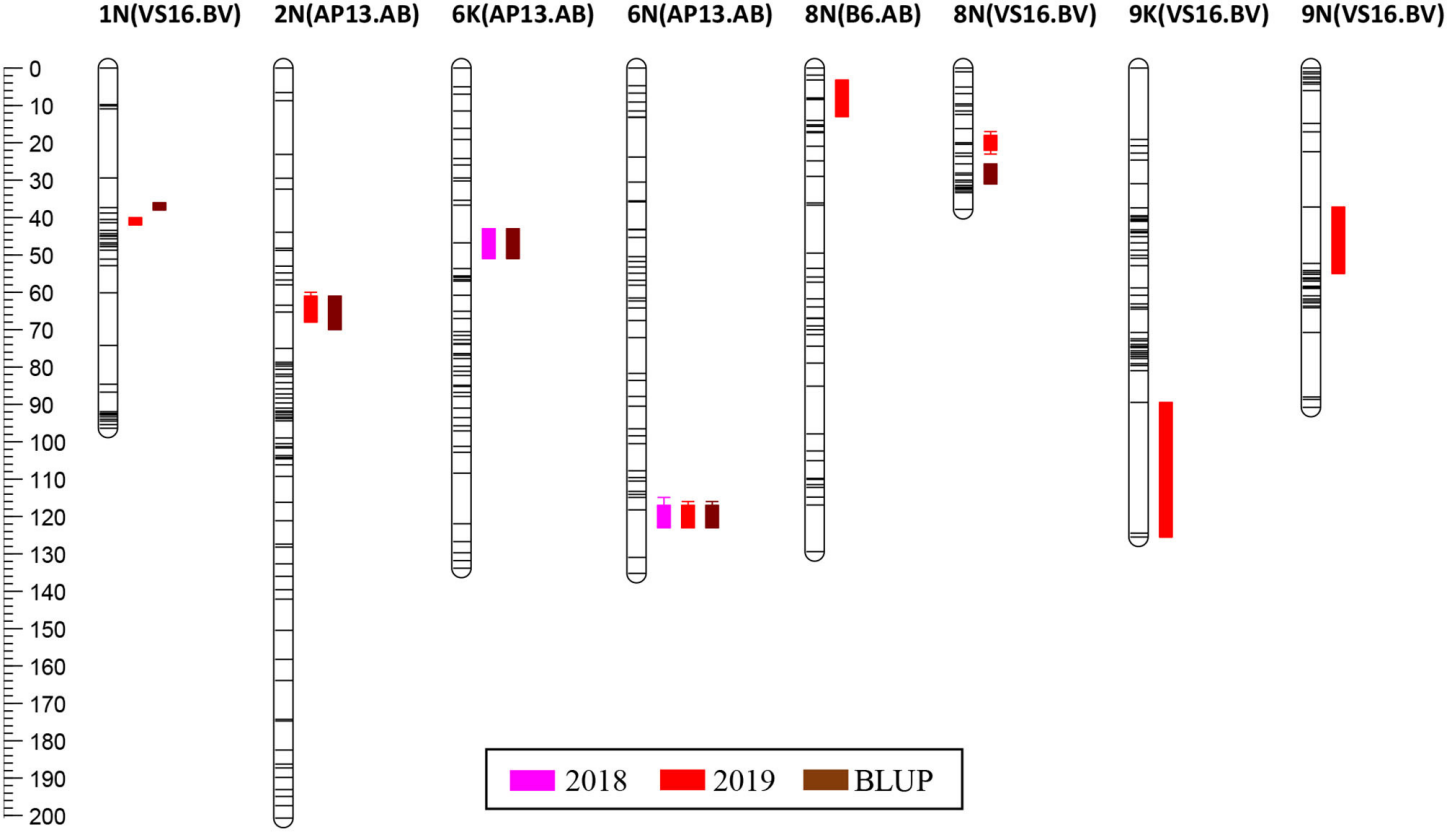
QTL positions on the genetic map. QTL are positioned at the right side of each LG; solid bars and whiskers on one or both ends represent coverage at LOD drop intervals of 1.0 and 2.0, respectively. QTL were mapped using LS means for each year and the BLUP value.

In the AB population, we found QTL in the AP13 map on LGs 2N (2019, BLUP), 6K (2018, BLUP), and 6N (2018, 2019, BLUP), and in the B6 map on LG 8N (2019, BLUP) (Table 4; Figure 4; Supplementary File 7). The range of the percentage of variance explained (PVE) by the QTL in the AB population was 6.2−11.4%. The QTL with the largest and most consistent effect across years in the AB population was found on LG 6N (Table 4); the marker most closely linked to this QTL is AB9342r (PVE: 8.4–11.5%). While we also found a QTL in the AB population on LG 6K, the 6N and 6K QTLs are not homoeologous.

**Table 4:** Biomass QTL detected using LS means value for each year and BLUP value across years (AB and BV) and planting date (BV).

**No**.	**Map-population**	**Biomass data**	**LG**	**Marker at LOD peak**	**Position (cM)**	**Position in *P. virgatum* V4.1**	**Left marker**	**Right marker**	**LOD**	**PVE (%)**	**Additive (g)**
1.	AP13.AB	2018, BLUP	6K	AB8348	46.8	12029478	–	–	4.49, 4.53	7.6,	−161.0,
										7.6	−124.2
2.	AP13.AB	2018, 2019, BLUP	6N	AB9342r	118.2	76759767	AB9333r	–	6.74, 5.02, 6.97	11.1,	−223.1,
										8.4,	−212.7,
										11.5	−149.9
3.	AP13.AB	2019, BLUP	2N	AB2789r	63.5	14410442	–	AB2809r	4.82, 4.46	8.1,	−187.8,
										7.5	−129.6
4.	B6.AB	2019	8N	AB11257	8.0	1365660	AB4784	AB11260	4.55	7.5	180.5
5.	VS16.BV.P1	2019	9K	BV30407	124.5	71035971	–	–	4.32	27.1	−272
6.	VS16.BV.P2	2019	1N	BV2509r	41.4	32302068	BV24860	–	3.17	9.2	−97.8
7.	VS16.BV.P2	2019	8N	BV23940r	20.4	10629137	BV24639r	BV24482	4.54	12.9	113.7
8.	VS16.BV.P2	2019	9N	BV29326r	52.3	63174050	–	BV26788	4.29	12.3	−118.3
9.	VS16.BV	BLUP	1N	BV2253r	37.3	8989747	–	–	3.17	6.6	−13.8
10.	VS16.BV	BLUP	8N	BV24791r	33.3	32644545	BV24582	–	3.90	8.1	72.6

*Map-population, AP13.AB AP13 map of the AB population, B6.AB B6 map of the AB population, VS16.BV.P1 VS16 map of the first set (63 progeny) of the BV population, VS16.BV.P2 VS16 map of the second set (151 progeny) of the BV population; LG linkage group; Left marker The most left-hand marker relative to the QTL peak with a LOD value at or higher than the threshold delineating the left-hand boundary of the QTL; Right marker The most right-hand marker relative to the QTL peak with a LOD value at or higher than the threshold delineating the QTL on the right-hand side; cM Centimorgan position; LOD logarithm of odds score; PVE percentage of trait variance explained by the QTL*.

For the BV population, QTL was identified only in the VS16 map on LGs 1N (2019, BLUP), 8N (2019, BLUP), 9K (2019), and 9N (2019) (Table 4; Figure 4; Supplementary File 7). The range of the percentage of variance explained (PVE) by the QTL in the BV population was 6.6–27.1%. However, the value of 27.1% for the 9K QTL identified in the VS16 map using the biomass data in 2019 is almost certainly an overestimation due to the small population size (a set of 63 BV progeny planted in 2017). The largest effect QTL in the BV population was located on LG 8N and most closely associated with marker BV23939r in 2019 (PVE: 12.3%), and markers BV24582 and BV24791r in the BLUP analysis (PVE: 8.1%) (Table 4). The 2019 and BLUP biomass peaks are non-overlapping (Figure 4).

## Discussion

### Genetic Map Characteristics

In this study, we compared genetic maps generated using genotyping-by-sequencing in two inter-ecotypic switchgrass F_1_ populations: lowland-coastal (AP13 × B6) and coastal-upland (B6 × VS16). The two populations share a common parent, B6, which is a winter and non-dormant coastal genotype. After the removal of non-hybrids and progeny with >20% of missing data, the number of progeny used for mapping was 263 and 267 for the AP13 and B6 maps, respectively, in the AB population, and 213 and 214 for the B6 and VS16 maps, respectively, in the BV population. The size of the mapping population is important to correctly order markers that are closer than 2–5 cM (Gardner et al., 2014) and to improve map resolution (Poland et al., 2012). The moderate number of progeny used in this study is comparable to the numbers of progeny used in many linkage mapping studies, which typically include around 80-200 progeny (Poland et al., 2012; Serba et al., 2013; Gardner et al., 2014; Li et al., 2014; Lowry et al., 2015).

We used genotyping-by-sequencing to identify variants for genetic mapping. More reads were generated for the BV population (2.4 B) compared to the AB population (2.2 B). Furthermore, a higher percentage of BV reads (73.26%) aligned to the V4.1 reference genome than AB reads (65.12%). Both are likely caused by the overall higher quality of the GBS libraries for the BV compared to the AB population.

The average allele count per site for the pooled reads was 519 and 536 in AB and BV, respectively. There was a considerable variation in read depth across individuals, however many progenies had a read depth ≥8X at individual SNP loci, which is the threshold value we used for genotyping. It has previously been shown that 8X coverage is sufficient to easily differentiate homozygous and heterozygous loci (Qi et al., 2018, 2021). Qi et al. (2018) also found that the optimal read number per sample when using the enzyme combination *Pst*I/*Msp*I was 2 million. We noted an exponential relationship between the number of reads per sample and the percent of missing data after scoring SNPs at 8X (Supplementary Figure 4). In both populations, all samples with <1.2 M reads had >20% missing data. For samples with 1.2–1.5-M reads, the 20% missing data threshold was reached for 50% of AB samples and 91% of BV samples. In contrast, only 1.7% of samples in the AB population and 4.1% of samples in the BV population with ≥2-M read had >20% of missing data at our scoring threshold of 8X. This suggests that the pool of *Pst*I/*Msp*I fragments obtained for each sample was smaller in the AB population than in the BV population, potentially due to a tighter size selection in the former.

A total of 6M raw SNP variants were identified in the BV population compared to 2.5M in the AB population. After filtering, this number declined to 27,517 SNPs that differentiated the B6 and VS16 parents and 14,816 SNPs that differentiated the AP13 and B6 parents. The higher number of SNPs in the BV compared to the AB population is a reflection of the greater Euclidean distance between the coastal B6 and upland VS16 genotypes (0.917) than between the coastal B6 and lowland AP13 genotypes (0.880), and will also be affected by the level of heterozygosity within the parents, and the size and read depth of the sequenced GBS fragment pool.

The SNP sets included only 398 markers in the AB population and 758 in the BV population that were heterozygous in both parents. To generate solid high-density maps, we, therefore, decided on using a pseudo-testcross design, mapping only markers that were present in the heterozygous condition in one parent and homozygous in the other parent. This led to four linkage maps with a total number of 2,410 SNPs in the AB population and 3,107 SNPs in the BV population. Projecting the GBS reads corresponding to the mapped SNPs on the switchgrass reference genome V5.1 through BLASTN showed that, in the AB population, 99.40% of markers for the AP13 map and 98.24% for the B6 map, and in the BV population, 97.95% of markers for the B6 map and 98.50% for the VS16 map located to the correct chromosome. Fiedler et al., 2015 found ~60% of markers in their severely distorted map to align to the same chromosome when *P. virgatum* V1.1 was used for alignment. As also noted by Qi et al. (2021), the high synteny seen in our study suggests a tremendous improvement of switchgrass assembly V5.1 compared to earlier versions since we only have 1.12% and 1.77% of markers in the AB and BV populations, respectively, that were not aligned to the corresponding chromosome. Conversely, the high levels of colinearity also demonstrate the robustness of our genetic maps.

A comparison of the genetic maps with the genome assembly also showed that not all maps displayed complete chromosome coverage. An overall lack of markers in the centromeric region was expected because the use of the methylation-sensitive restriction enzyme *Pst*I leads to an enrichment of GBS tags in genic regions. Other regions missing from the genetic maps, such as the proximal region of chromosome 1K in the B6 map in both the AB and BV populations appear to be caused by a lack of markers that are heterozygous in B6. This is demonstrated by the number of SNPs identified in the three parents. Without considering the segregation of those markers in the progeny, 23 markers were heterozygous in the B6 parent in the first 12 Mb of chromosome 1K, and 333 were homozygous. Another example is the proximal 19 Mb of chromosome 9N, which has 23 heterozygous markers and 635 homozygous markers in VS16. Not surprisingly, this region of chromosome 9N is lacking from the VS16 map. Although switchgrass is an obligate outcrosser, large stretches of homozygosity could occur if specific haplotypes are present at a sufficiently high frequency in the population.

Even though more markers were included in the BV map, the total map length was shorter for both parental maps in the BV compared to the AB population. This is due to the lower average amount of recombination per Mb in the BV maps (1.37 and 1.39 cM/Mb for the B6 and VS16 maps, respectively) compared to AB maps (3.08 and 2.34 cM/Mb for the AP13 and B6 maps, respectively). The recombination rate (cM/Mb) was calculated only for those linkage groups that showed high coverage of the corresponding chromosome in the genome assembly, and for which the total coverage varied by <10% between the maps. Although the B6 parent was common to both crosses, the recombination rate of the B6 parent varied significantly in the two mapping populations (*p* = 0.001). It is well known that male and female recombination rates can vary, with both the size and direction of the effect being species-dependent (de Vicente and Tanksley, 1991; Giraut et al., 2011; Stapley et al., 2017; Luo et al., 2019). It seems unlikely that the differences in recombination rates observed here between AP13, B6, and VS16 simply reflect genotypic and/or sex effects. Indeed, a mapping study conducted in F_1_ progeny from a cross between AP13 and VS16 discerned no significant differences in length between the two parental maps (Serba et al., 2013). One notable difference between the AB and BV maps that needs to be given further consideration is the high level of segregation distortion seen in both AB maps. Transmission ratio distortion can introduce spurious linkages, biased estimates of recombination fractions, or incorrect marker orders, leading to map inflation (Okada et al., 2010).

### Segregation Distortion

Inter- and intraspecific crosses often lead to a distortion of segregation ratios in the hybrid progeny (Faris et al., 1998; Virk et al., 1998; Mano et al., 2005; Törjék et al., 2006). Segregation distortion is a deviation of segregation ratios from the expected Mendelian fractions (Daniel and Yaakov, 1986; Lyttle, 1991) that may result from competition among gametes or from the abortion of gametes or zygotes. Competition among gametes may occur because of gametophyte genes expressed during postmeioticosis of the microspore and pollen development in angiosperms (Lyttle, 1991; Mascarenhas, 1992). Genetic differences among pollen may lead to gamete competition and selection, which results in non-random fertilization, while hybrid sterility genes can cause abortion of a specific gamete or zygote genotypes (Mascarenhas, 1992). Because we are using a pseudo-testcross design to conduct genetic mapping in F_1_ progeny from inter-ecotypic crosses, we are assessing recombination within the parental lines, which are expected to carry alleles from only a single ecotype. If segregation distortion is seen in only one parental map, it may be caused by prezygotic factors, such as non-random gamete production or the presence of a factor that provides a competitive advantage to these gametes during fertilization. If so, the distortion would be expected to also be observed if that same parent was used as the same-sex parent in a different cross. While the B6 parent was common to both crosses, it was used as the male parent in the AB cross and the female parent in the BV cross. However, recombination and segregation distortion have previously been assessed in AP13 when used as the female parent (as in the AB population) and in VS16 when used as the male parent (as in the BV population) in a genetic mapping study in F_1_ progeny from an AP13 × VS16 cross (Serba et al., 2013). The VS16 paternal map generated in the AP13 × VS16 population showed distortion on chromosomes 1N (previously Ia), 3N (previously IIIb), and 7N (previously VIIb). The VS16 paternal map in the BV population similarly showed parent-specific distortion on chromosome 7N. Distortion on 7N has also been observed in the paternal map of a lowland Alamo genotype (Okada et a. 200), and has putatively been attributed to the presence of a self-incompatibility (SI) locus based on synteny to a region of rye carrying the Z locus. SI loci can cause a failure of fertilization by the affected pollen and hence lead to marker distortion in the male maps.

Very little distortion was seen in the AP13 maternal map in the AP13 × VS16 population generated by Serba and colleagues (Serba et al., 2013). In contrast, extensive segregation distortion was observed on most chromosomes in both the maternal AP13 and paternal B6 maps generated in the AB population. The F_1_ progeny in this cross are lowland-coastal inter-ecotypic hybrids, and we hypothesize the existence of a zygotic or post-zygotic interaction between alleles of the two ecotypes that, globally, leads to selection for higher levels of heterozygosity. No such selection was seen in the BV population, despite that the F_1_ is also inter-ecotypic hybrids, albeit between an upland and coastal genotype, or in the AP13 × VS16 population, where F_1_ progeny are upland-lowland hybrids. Because the genetic similarity is higher between AP13 and B6 than between VS16 and B6 (Table 2), zygotes with higher levels of homozygosity may have reduced fitness or, conversely, heterozygosity results in heterosis. However, extensive segregation distortion was not previously identified in F_1_ progeny of the AP13 × VS16 cross, despite that AP13 and VS16 were shown here to be more genetically similar than either AP13 and B6 or VS16 and B6. It should be noted that the Euclidean genetic distances calculated between the parents based on the GBS data do not follow the patterns of genetic similarity previously observed at the population level. Recent studies showed that there is a significant population structure in switchgrass with the upland genotypes largely grouping into one subpopulation “C1” (which comprises VS16) and the lowland genotypes grouping into two subpopulations, “C2” (comprising AP13) and “C3” (comprising B6) (Bahri et al., 2018, 2020). Analyses based on a small subset of genes (Bahri et al., 2018, 2020) as well as at the whole-genome level (B.A. Bahri, P. Qi, and K.M. Devos, unpublished data) indicated the greatest genetic diversity and lowest gene flow between C1 and C2. Upland accessions are commonly octoploid, while lowland accessions are tetraploid. Gene flow, however, can occur freely between upland and lowland genotypes of the same ploidy level. The upland genotype VS16 is tetraploid, and this may explain the discrepancy between the levels of divergence observed between C1 and C2 at the population level and the genotypic level when the genotypes being compared have the same ploidy.

The change in direction of the distortion seen in the AB maps (Supplementary Figures 3a,b) corresponds to the transition between marker blocks that were originally in a different linkage phase. This strong grouping of markers by linkage phase would be expected if large regions of a chromosome in the switchgrass V5.1 genome assembly were phased so that the reference alleles in the AP13 parent formed one haplotype, and the alternate alleles a second haplotype. However, while a similar marker grouping is seen in the B6 map of the AB population, it is not observed in the BV maps. In the latter maps, markers with and without the suffix “r” are interspersed, as would be expected from non-phased chromosomes in a genome assembly. Furthermore, a comparison of the SNP markers that were the input for the PhaseI map generation and those that were built into the map showed that, across large regions, only markers that were in the same linkage phase were incorporated. We examined whether JoinMap had failed to link those markers, perhaps because of the high level of segregation distortion. However, we saw the same clustering of markers by linkage phase when maps were generated using MAPMAKER after manually having standardized all markers to the same linkage phase. We hypothesize that the elimination of markers may have been caused by a greater impact of the effect of scoring errors due to the segregation distortion.

### Biomass QTL

Several studies have been reported on the QTL mapping of biomass yield in switchgrass (Lowry et al., 2015; Serba et al., 2015; Chang et al., 2016; Milano et al., 2016; Taylor et al., 2019). Because biomass yield is a complex trait controlled by multiple loci, and different studies employ different populations grown under different environmental conditions, we expect to see variations in the mapping. In our study, QTL were found on four chromosomes in each of the AB and BV populations. It is possible that the segregation distortion affected the power for detecting QTL. However, segregation distortion is not expected to lead to false positives or impact the location and effects of QTL (Zhang et al., 2010). Both populations had QTL on chromosome 8N, although the QTL were not overlapping. The QTL on 8N identified in the BV population in the 2019 and across years (BLUP) analyses also did not overlap, probably because of the different biomass distribution across years for the two sets of progeny that were planted in consecutive years. A number of biomass QTL have previously been mapped in an AP13 map generated in an AP13 × VS16 cross, including on chromosomes 6K (previously VIa) (Serba et al., 2015). However, no QTL was identified by Serba and colleagues on chromosome 6N (previously VIb), which houses the largest-effect QTL in our study. Similarly, Serba et al. (2015) identified multiple biomass QTL in the VS16 map, one of which localized to the same chromosome (9K, previously IXb) as one of the QTL in our study. Unfortunately, it is unclear whether the chromosome 6K and 9K QTL in the two studies colocalize because the markers in the Serba et al. (2015) study have not been positioned onto the genome assembly. It should be noted that the biomass QTL mapped here does not represent variation between the parents, but rather a variation that exists within each parent for biomass yield. All F_1_ are inter-ecotype hybrids, and the biomass variation seen between the F_1_ progeny depends on which allele is contributed from each of the parents. We would expect to see QTL of much larger effects when mapping in the F_2_ generation of an inter-ecotypic cross.

## Conclusions

We have successfully produced linkage maps for all parents in both populations and conducted QTL mapping of biomass yield. A comparison of the total number of variants in the raw data showed that the population derived from the cross between the coastal and upland ecotypes contained more polymorphisms than the cross between the lowland and coastal ecotypes. This was supported by the higher genetic distance between the B6 and VS16 parents compared to the AP13 and B6 parents, although other factors such as heterozygosity within parents, and the size and sequence depth of the GBS fragment pool also play a role. Different from the expectation that a population derived from more diverged parents (BV population) would have more distorted markers due to possible reproductive barriers, the less diverged AB population had more distorted markers. The higher distortion in AB could be due to lower compatibility between the lowland AP13 and the newly classified coastal ecotype B6. Zygotic or post-zygotic selection for increased levels of heterozygosity suggests reduced fitness of homozygotes or heterosis conferred by heterozygous loci in the coastal × lowland hybrids. Since switchgrass improvement is based on increasing favorable allele frequencies through recurrent selection, the transmission bias within individuals and loci needs to be accounted for as this may affect the genetic gain if the favorable alleles are distorted. Finally, biomass QTL can be used in our breeding program to screen for potential high biomass yield progeny.

## Data Availability Statement

All data generated or analyzed during this study are included in this published article (and its Supplementary Information files). Variant files and their metadata are publicly available at Figshare.com (https://doi.org/10.6084/m9.figshare.13120271).

## Author Contributions

RR conducted all experiments, collected, analyzed the data, and wrote the manuscript. PQ conducted the PhaseII mapping and assisted with the QTL analysis. KD supervised the GBS and linkage mapping works and assisted with data interpretation and manuscript writing. AM supervised the research, edited, and revised the manuscript. All authors read and approved the final manuscript.

## Funding

The Malaysian Rubber Board funded RR's salary and bench fees. Partial funding was provided by the Center for Bioenergy Innovation, a US Department of Energy Research Center supported by the Office of Biological and Environmental Research in the DOE Office of Science, and for biomass harvesting equipment and technical staff support.

## Conflict of Interest

The authors declare that the research was conducted in the absence of any commercial or financial relationships that could be construed as a potential conflict of interest. The reviewer HY declared a shared affiliation with the authors to the handling editor at the time of the review.

## Publisher's Note

All claims expressed in this article are solely those of the authors and do not necessarily represent those of their affiliated organizations, or those of the publisher, the editors and the reviewers. Any product that may be evaluated in this article, or claim that may be made by its manufacturer, is not guaranteed or endorsed by the publisher.
