# Supplementary material for: Genotyping-by-Sequencing and QTL Mapping of Biomass Yield in Two Switchgrass F1 Populations (Lowland x Coastal and Coastal x Upland)
Source: Front Plant Sci. 2022 May 19;13:739133. doi: 10.3389/fpls.2022.739133 (PMC9162799; doi:10.3389/fpls.2022.739133)
Supplement: Supplementary file 7 [file Data_Sheet_1.docx]

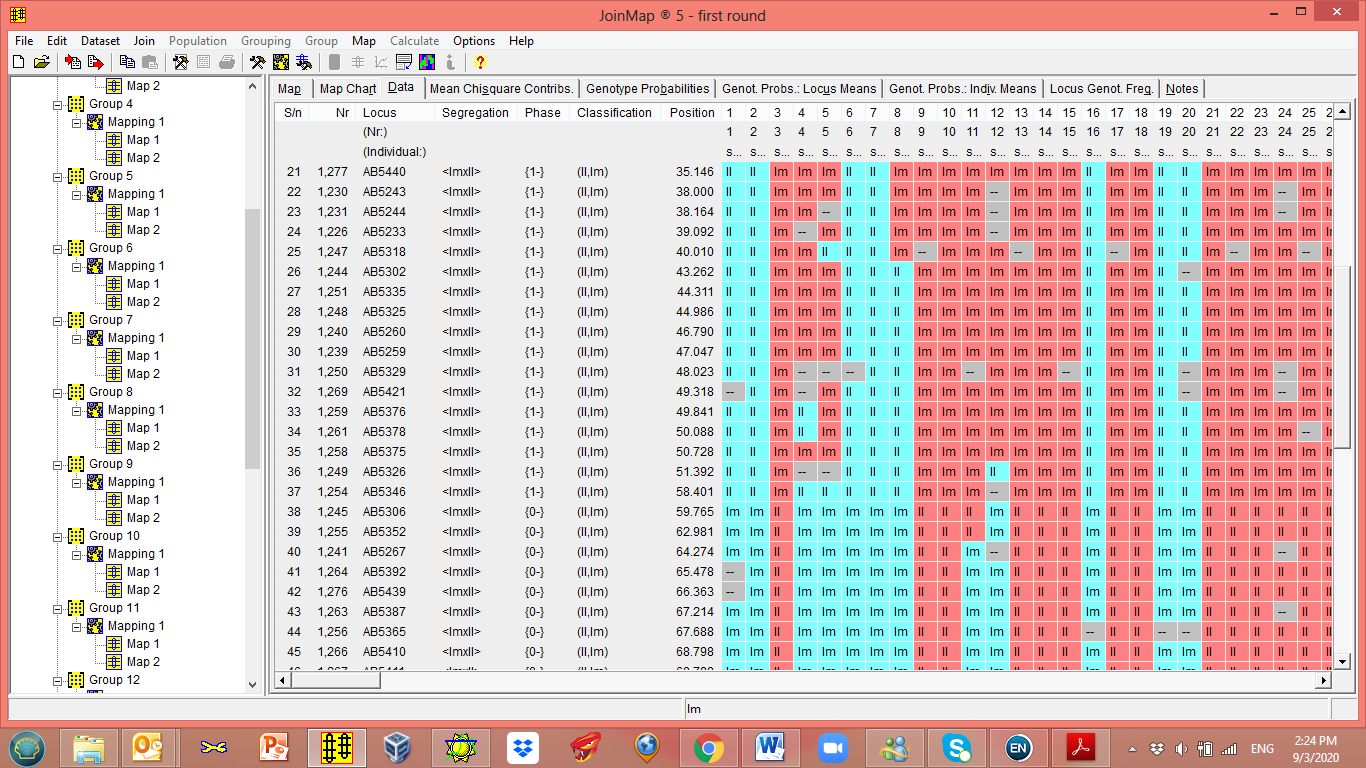


**(A)**

| Marker | cM | 1 | 2 | 3 | 4 | 5 | 6 | 7 | 8 | 9 | 10 | 11 | 12 | 13 | 14 | 15 | 16 | 17 | 18 | 19 | 20 | 21 | 22 | 23 | 24 | 25 |
| --- | --- | --- | --- | --- | --- | --- | --- | --- | --- | --- | --- | --- | --- | --- | --- | --- | --- | --- | --- | --- | --- | --- | --- | --- | --- | --- |
| AB5440 | 35.146 | ll | ll | lm | lm | lm | ll | ll | lm | lm | lm | lm | lm | lm | lm | lm | ll | lm | lm | ll | ll | lm | lm | lm | lm | lm |
| AB5243 | 38 | ll | ll | lm | lm | lm | ll | ll | lm | lm | lm | lm | -- | lm | lm | lm | ll | lm | lm | ll | ll | lm | lm | lm | -- | lm |
| AB5244 | 38.164 | ll | ll | lm | lm | -- | ll | ll | lm | lm | lm | lm | -- | lm | lm | lm | ll | lm | lm | ll | ll | lm | lm | lm | -- | lm |
| AB5233 | 39.092 | ll | ll | lm | -- | lm | ll | ll | lm | lm | lm | lm | -- | lm | lm | lm | ll | lm | lm | ll | ll | lm | lm | lm | lm | lm |
| AB5318 | 40.01 | ll | ll | lm | lm | ll | ll | ll | lm | -- | lm | lm | lm | -- | lm | lm | ll | -- | lm | ll | ll | lm | -- | lm | lm | -- |
| AB5302 | 43.262 | ll | ll | lm | lm | lm | ll | ll | ll | lm | lm | lm | lm | lm | lm | lm | ll | lm | lm | ll | -- | lm | lm | lm | lm | lm |
| AB5335 | 44.311 | ll | ll | lm | lm | lm | ll | ll | ll | lm | lm | lm | lm | lm | lm | lm | ll | lm | lm | ll | ll | lm | lm | lm | lm | lm |
| AB5325 | 44.986 | ll | ll | lm | lm | lm | ll | ll | ll | lm | lm | lm | lm | lm | lm | lm | ll | lm | lm | ll | ll | lm | lm | lm | lm | lm |
| AB5260 | 46.79 | ll | ll | lm | lm | lm | ll | ll | ll | lm | lm | lm | lm | lm | lm | lm | ll | lm | lm | ll | ll | lm | lm | lm | lm | lm |
| AB5259 | 47.047 | ll | ll | lm | lm | lm | ll | ll | ll | lm | lm | lm | lm | lm | lm | lm | ll | lm | lm | ll | ll | lm | lm | lm | lm | lm |
| AB5329 | 48.023 | ll | ll | lm | -- | -- | -- | ll | ll | lm | lm | -- | lm | lm | lm | -- | ll | lm | lm | ll | -- | lm | lm | lm | -- | lm |
| AB5421 | 49.318 | -- | ll | lm | -- | lm | ll | ll | ll | lm | lm | lm | lm | lm | lm | lm | ll | lm | lm | ll | -- | lm | lm | lm | -- | lm |
| AB5376 | 49.841 | ll | ll | lm | ll | lm | ll | ll | ll | lm | lm | lm | lm | lm | lm | lm | ll | lm | lm | ll | ll | lm | lm | lm | lm | lm |
| AB5378 | 50.088 | ll | ll | lm | ll | lm | ll | ll | ll | lm | lm | lm | lm | lm | lm | lm | ll | lm | lm | ll | ll | lm | lm | lm | lm | -- |
| AB5375 | 50.728 | ll | ll | lm | lm | lm | ll | ll | ll | lm | lm | lm | lm | lm | lm | lm | ll | lm | lm | ll | ll | lm | lm | lm | lm | lm |
| AB5326 | 51.392 | ll | ll | lm | -- | -- | ll | ll | ll | lm | lm | lm | ll | lm | lm | lm | ll | lm | lm | ll | ll | lm | lm | lm | lm | lm |
| AB5346 | 58.401 | ll | ll | lm | ll | ll | ll | ll | ll | lm | lm | lm | -- | lm | lm | lm | ll | lm | lm | ll | ll | lm | lm | lm | lm | lm |
| AB5306r | 59.765 | ll | ll | lm | ll | ll | ll | ll | ll | lm | lm | lm | ll | lm | lm | lm | ll | lm | lm | ll | ll | lm | lm | lm | lm | lm |
| AB5352r | 62.981 | ll | ll | lm | ll | ll | ll | ll | ll | lm | lm | lm | ll | lm | lm | lm | ll | lm | lm | ll | ll | lm | lm | lm | lm | lm |
| AB5267r | 64.274 | ll | ll | lm | ll | ll | ll | ll | ll | lm | lm | ll | -- | lm | lm | lm | ll | lm | lm | ll | ll | lm | lm | lm | -- | lm |
| AB5392r | 65.478 | -- | ll | lm | ll | ll | ll | ll | ll | lm | lm | ll | ll | lm | lm | lm | ll | lm | lm | ll | ll | lm | lm | lm | lm | lm |
| AB5439r | 66.363 | -- | ll | lm | ll | ll | ll | ll | ll | lm | lm | ll | ll | lm | lm | lm | ll | lm | lm | ll | ll | lm | lm | lm | lm | lm |
| AB5387r | 67.214 | ll | ll | lm | ll | ll | ll | ll | ll | lm | lm | ll | ll | lm | lm | lm | ll | lm | lm | ll | ll | lm | lm | lm | -- | lm |
| AB5365r | 67.688 | ll | ll | lm | ll | ll | ll | ll | ll | lm | lm | ll | ll | lm | lm | lm | -- | lm | lm | -- | -- | lm | lm | lm | lm | lm |
| AB5410r | 68.798 | ll | ll | lm | ll | ll | ll | ll | ll | lm | lm | ll | ll | lm | lm | lm | ll | lm | lm | ll | ll | lm | lm | lm | lm | Lm |

**(B)**

**Supplementary figure 1** Joinmap output (A) showing the linkage phase for each marker ({0-} or {1-}). This information was used to manually reverse genotypic scores (heterozygous to homozygous and *vice versa*) at marker loci with linkage phase {0-} to standardize the linkage phase in each linkage group for further analysis in MAPMAKER (B).


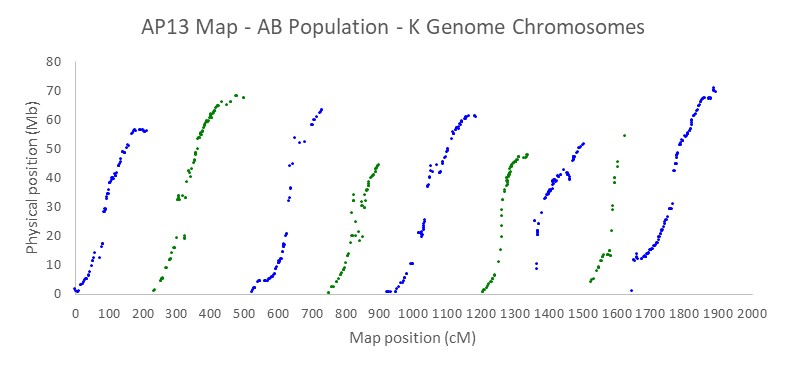


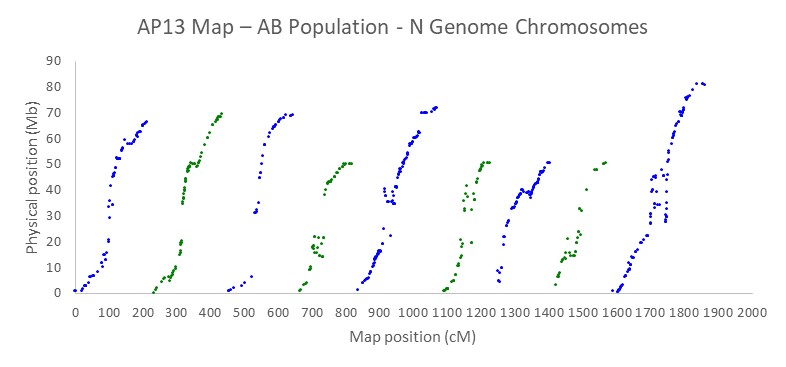


**Supplementary figure 2a** Colinearity of marker positions in genetic maps (x-axis) and switchgrass reference genome V5.1 (y-axis) across concatenated K and N subgenome chromosomes in AP13 map for AP13 x B6 population. Alternating colors were used to enhance the differentiation of neighboring chromosomes.

**
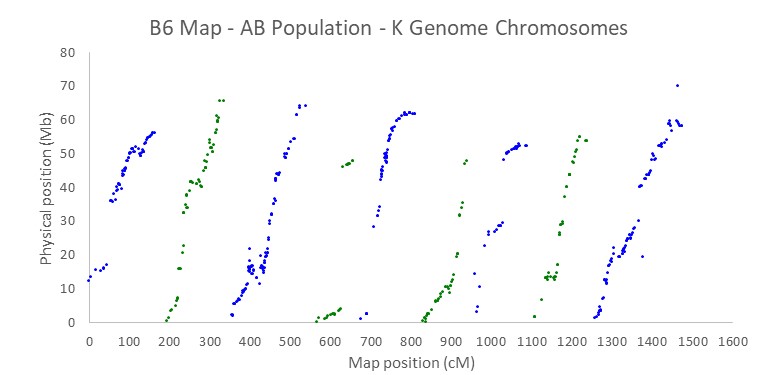
**


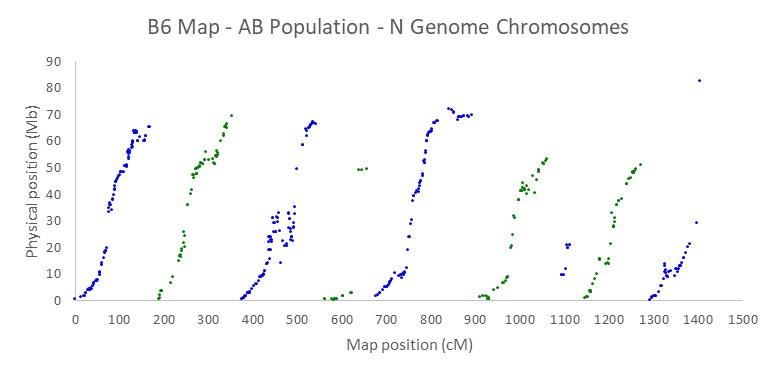


**Supplementary figure 2b** Colinearity of marker positions in genetic maps (x-axis) and switchgrass reference genome V5.1 (y-axis) across concatenated K and N subgenome chromosomes in B6 map for AP13 x B6 population. Alternating colors were used to enhance the differentiation of neighboring chromosomes.

**
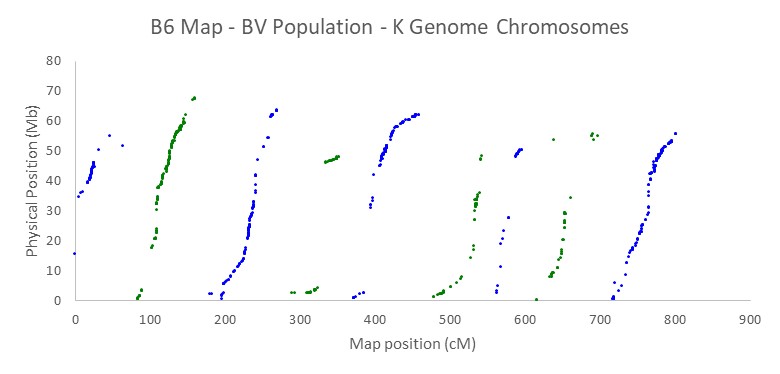
**


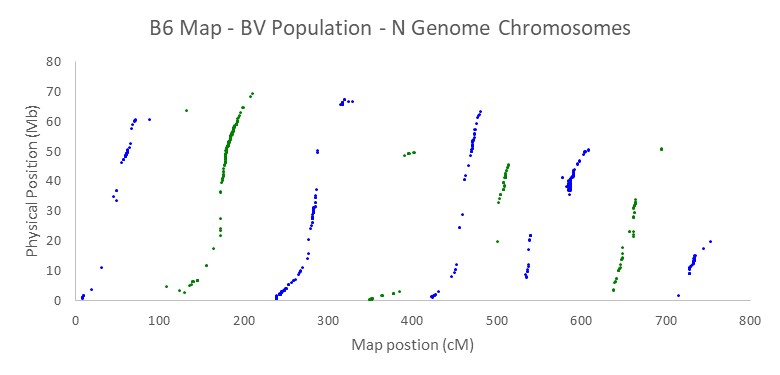


**Supplementary figure 2c** Colinearity of marker positions in the genetic maps (x-axis) and switchgrass reference genome V5.1 (y-axis) across concatenated K and N subgenome chromosomes in B6 map for B6 x VS16 population. Alternating colors were used to enhance the differentiation of neighboring chromosomes.

**
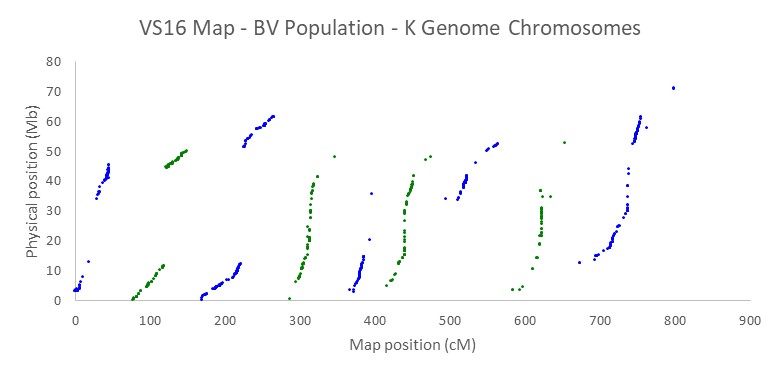
**


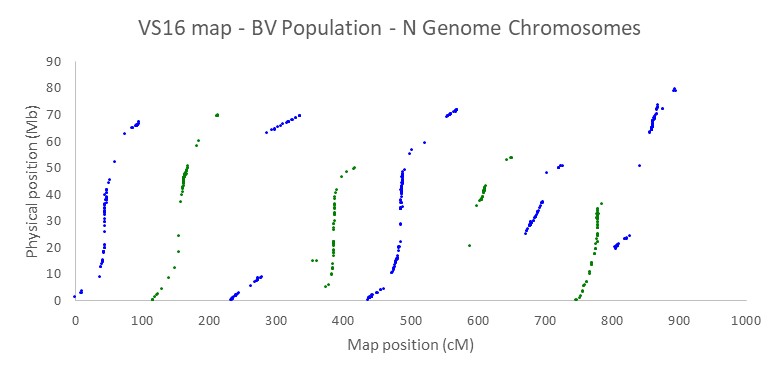


**Supplementary figure 2d** Colinearity of marker positions in the genetic maps (x-axis) and switchgrass reference genome V5.1 (y-axis) across concatenated K and N subgenome chromosomes in VS16 map for B6 x VS16 population. Alternating colors were used to enhance differentiation of neighboring chromosomes.

**
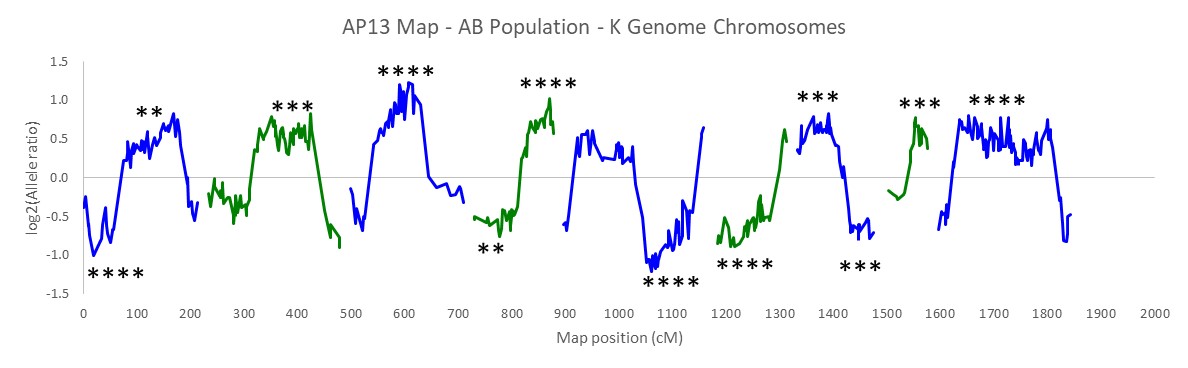
**


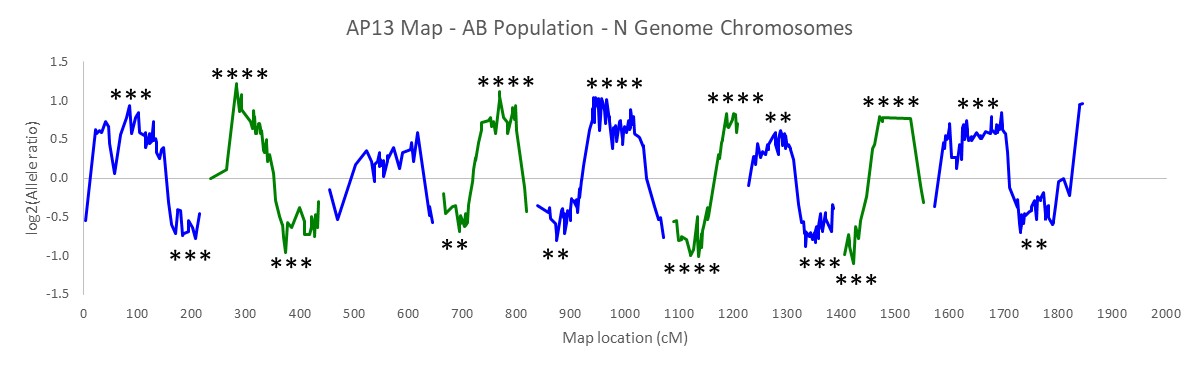


**Supplementary figure 3a** Graphs showing the log2 values of the A/H allele ratio across concatenated K and N subgenome chromosomes in AP13 maps of the AB population. Alternating colors were used to enhance the differentiation of neighboring chromosomes. Chromosomal regions consisting of at least seven consecutive markers with significant distortion are indicated with *, **, *** or **** signifying that the highest level of distortion, present in at least four consecutive markers, was significant at the 5%, 1%, 0.1% or 0.01% level, respectively.


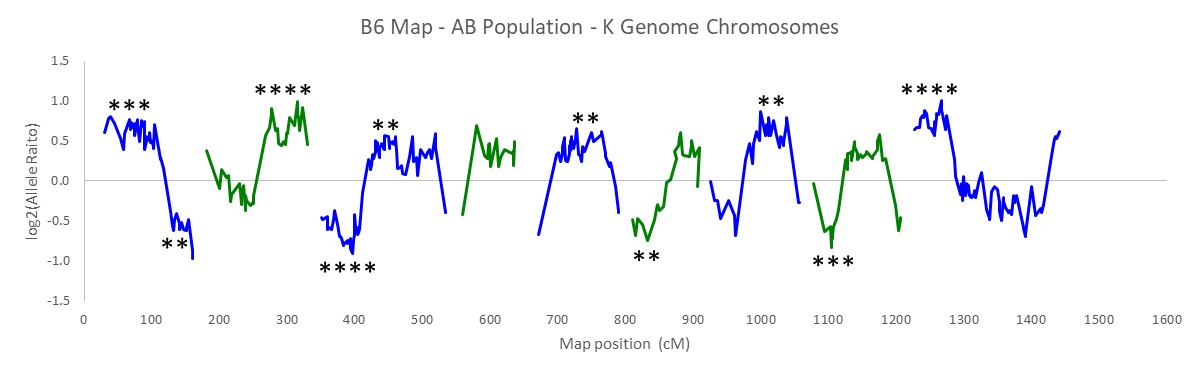


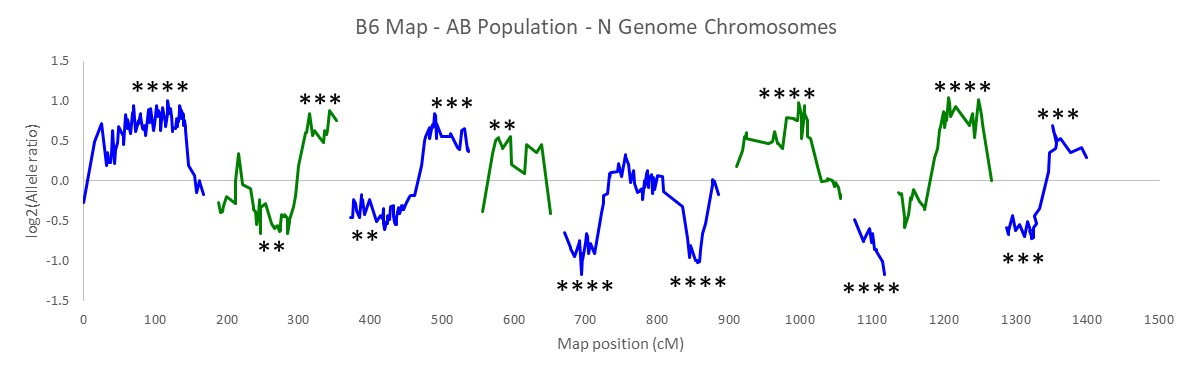


**Supplementary figure 3b** Graphs showing the log2 values of the A/H allele ratio across concatenated K and N subgenome chromosomes in B6 maps of the AB population. Alternating colors were used to enhance the differentiation of neighboring chromosomes. Chromosomal regions consisting of at least seven consecutive markers with significant distortion are indicated with *, **, *** or **** signifying that the highest level of distortion, present in at least four consecutive markers, was significant at the 5%, 1%, 0.1% or 0.01% level, respectively.


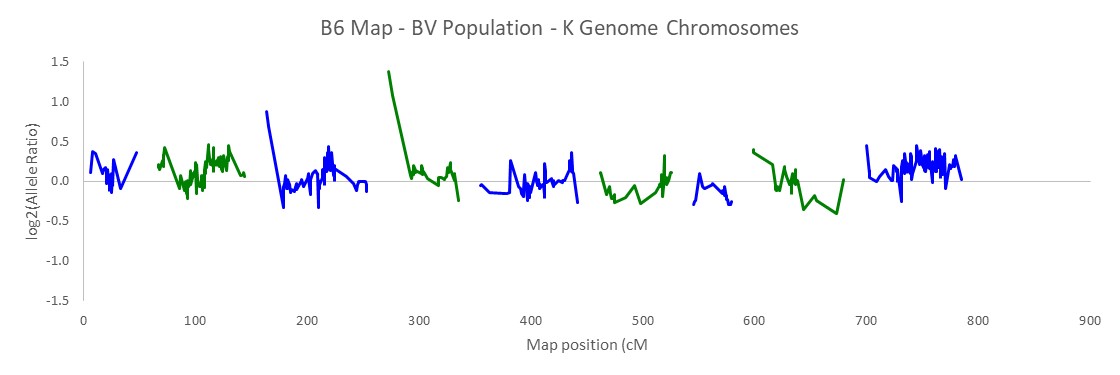


**
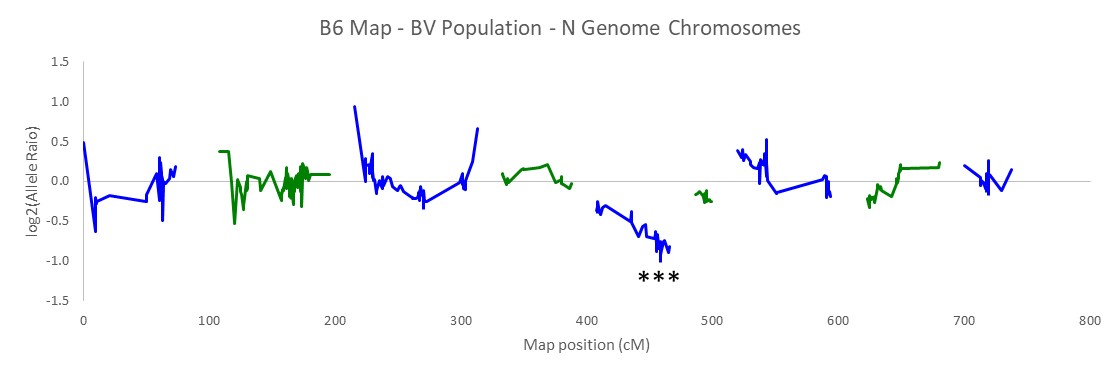
**

**Supplementary figure 3c** Graphs showing the log2 values of the A/H allele ratio across concatenated K and N subgenome chromosomes in B6 maps of the BV population. Alternating colors were used to enhance the differentiation of neighboring chromosomes. Chromosomal regions consisting of at least seven consecutive markers with significant distortion are indicated with *, **, *** or **** signifying that the highest level of distortion, present in at least four consecutive markers, was significant at the 5%, 1%, 0.1% or 0.01% level, respectively.


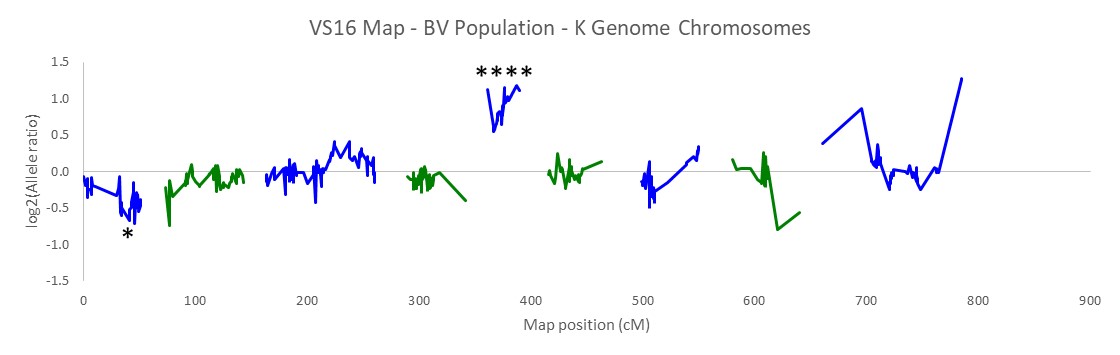


**
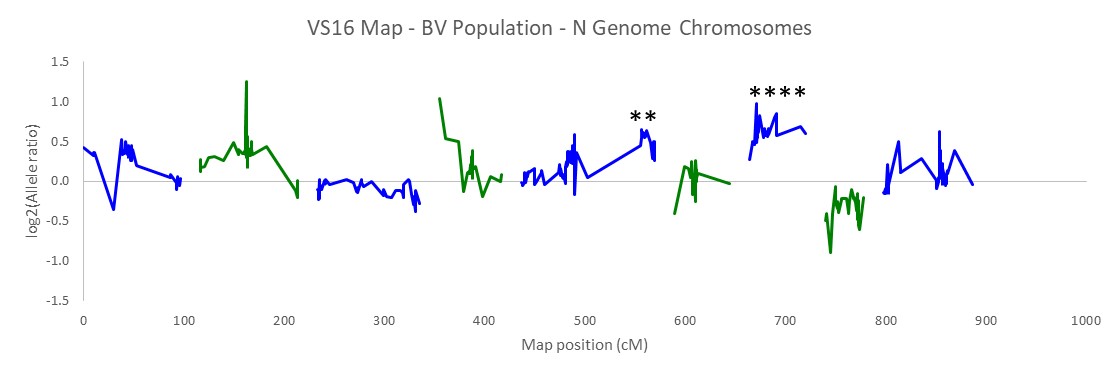
**

**Supplementary figure 3d** Graphs showing the log2 values of the A/H allele ratio across concatenated K and N subgenome chromosomes in VS16 maps of the BV population. Alternating colors were used to enhance differentiation of neighboring chromosomes. Chromosomal regions consisting of at least seven consecutive markers with significant distortion are indicated with *, **, *** or **** signifying that the highest level of distortion, present in at least four consecutive markers, was significant at the 5%, 1%, 0.1% or 0.01% level, respectively.

**
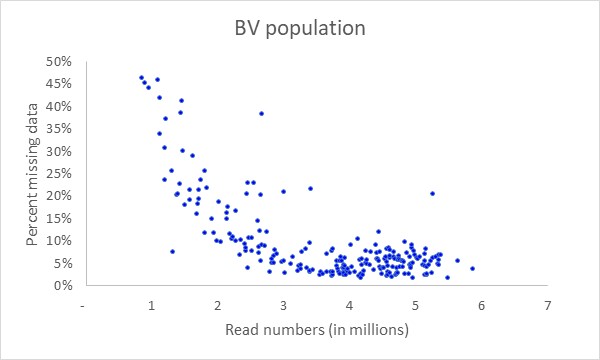

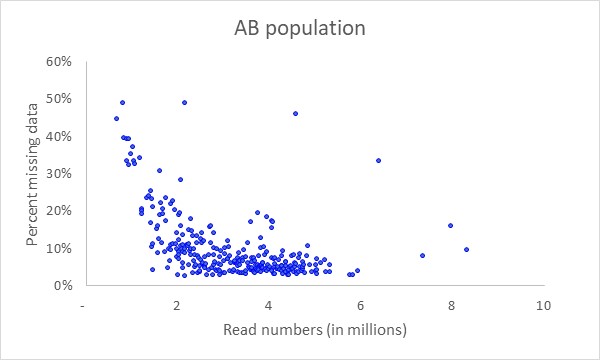
**

**Supplementary figure 4**. Scatterplot showing the relationship between read numbers and percent missing data at a scoring threshold of 8X in the AB and BV populations.
