# Supplementary material for: Genotyping-by-Sequencing and QTL Mapping of Biomass Yield in Two Switchgrass F1 Populations (Lowland x Coastal and Coastal x Upland)
Source: Front Plant Sci. 2022 May 19;13:739133. doi: 10.3389/fpls.2022.739133 (PMC9162799; doi:10.3389/fpls.2022.739133)
Supplement: Supplementary file 8 [file Presentation_1.PPTX]

## Slide 1
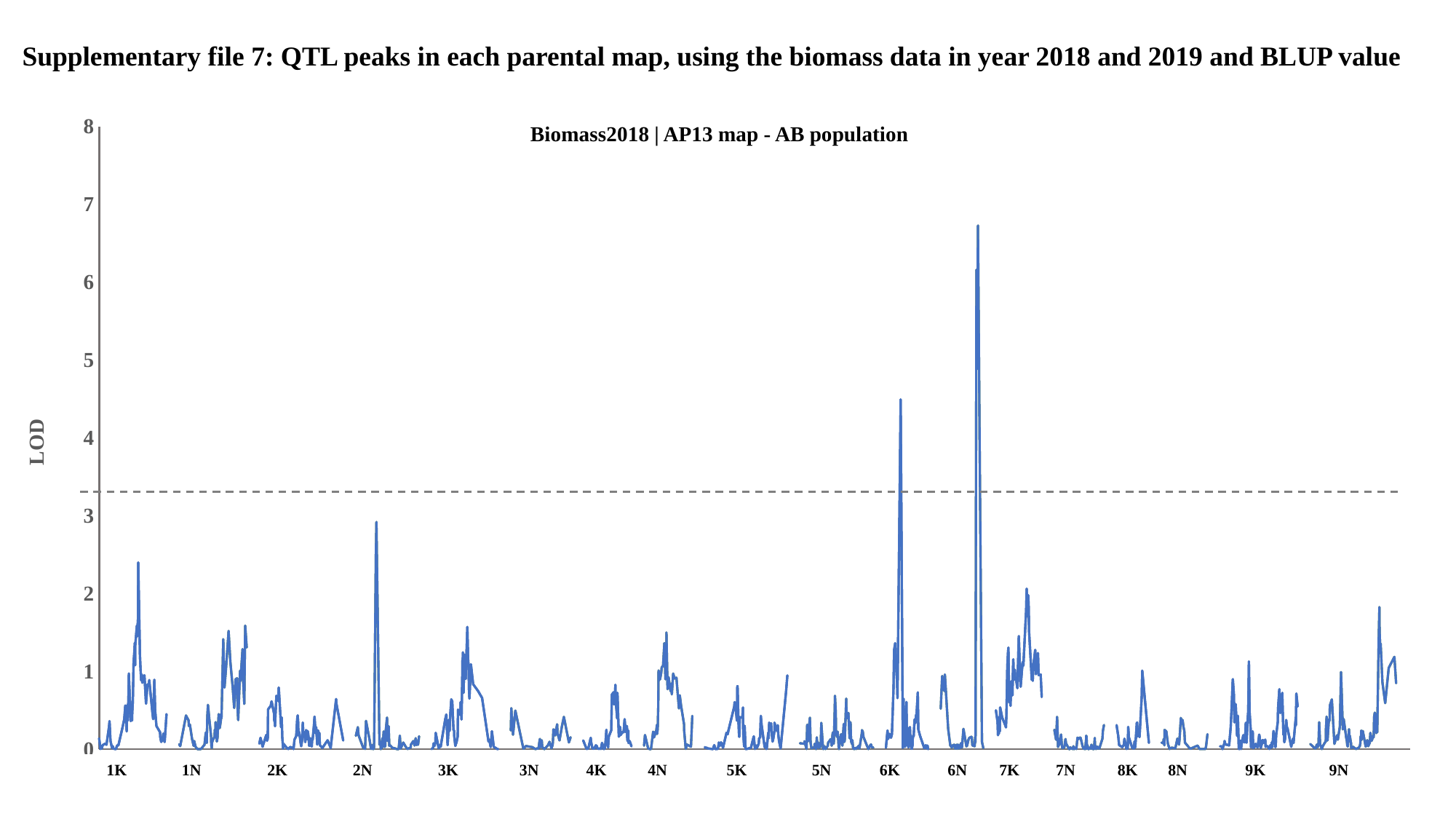

Supplementary file 7: QTL peaks in each parental map, using the biomass data in year 2018 and 2019 and BLUP value
### Chart
| Category | Biomass2018-AB | 1K | | | | 3K | 3N | 4K | 4N | 5K | 5N | 6K | 6N | 7K | 7N | 8K | 8N | 9K | 9N |
|---|---|---|---|---|---|---|---|---|---|---|---|---|---|---|---|---|---|---|---|Biomass2018 | AP13 map - AB population
1K
1N
2K
2N
3K
3N
4K
4N
5K
5N
6K
6N
7K
7N
8K
8N
9K
9N

## Slide 2
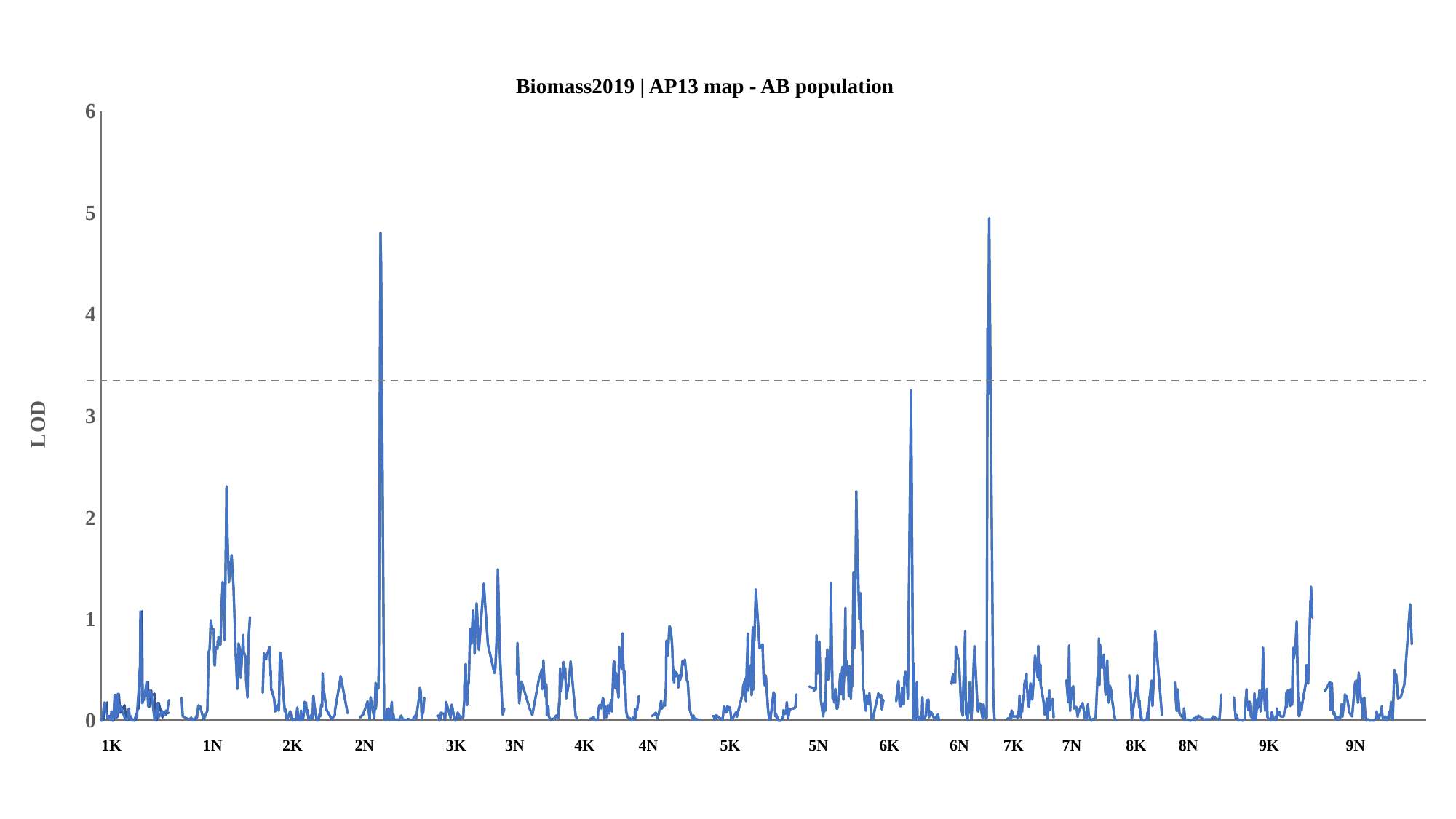

Biomass2019 | AP13 map - AB population
### Chart
| Category | | 1K | 1N | 2K | 2N | 3K | 3N | 4K | 4N | 5K | 5N | 6K | 6N | 7K | 7N | 8K | 8N | 9K | 9N |
|---|---|---|---|---|---|---|---|---|---|---|---|---|---|---|---|---|---|---|---|
1K
1N
2K
2N
3K
3N
4K
4N
5K
5N
6K
6N
7K
7N
8K
8N
9K
9N

## Slide 3
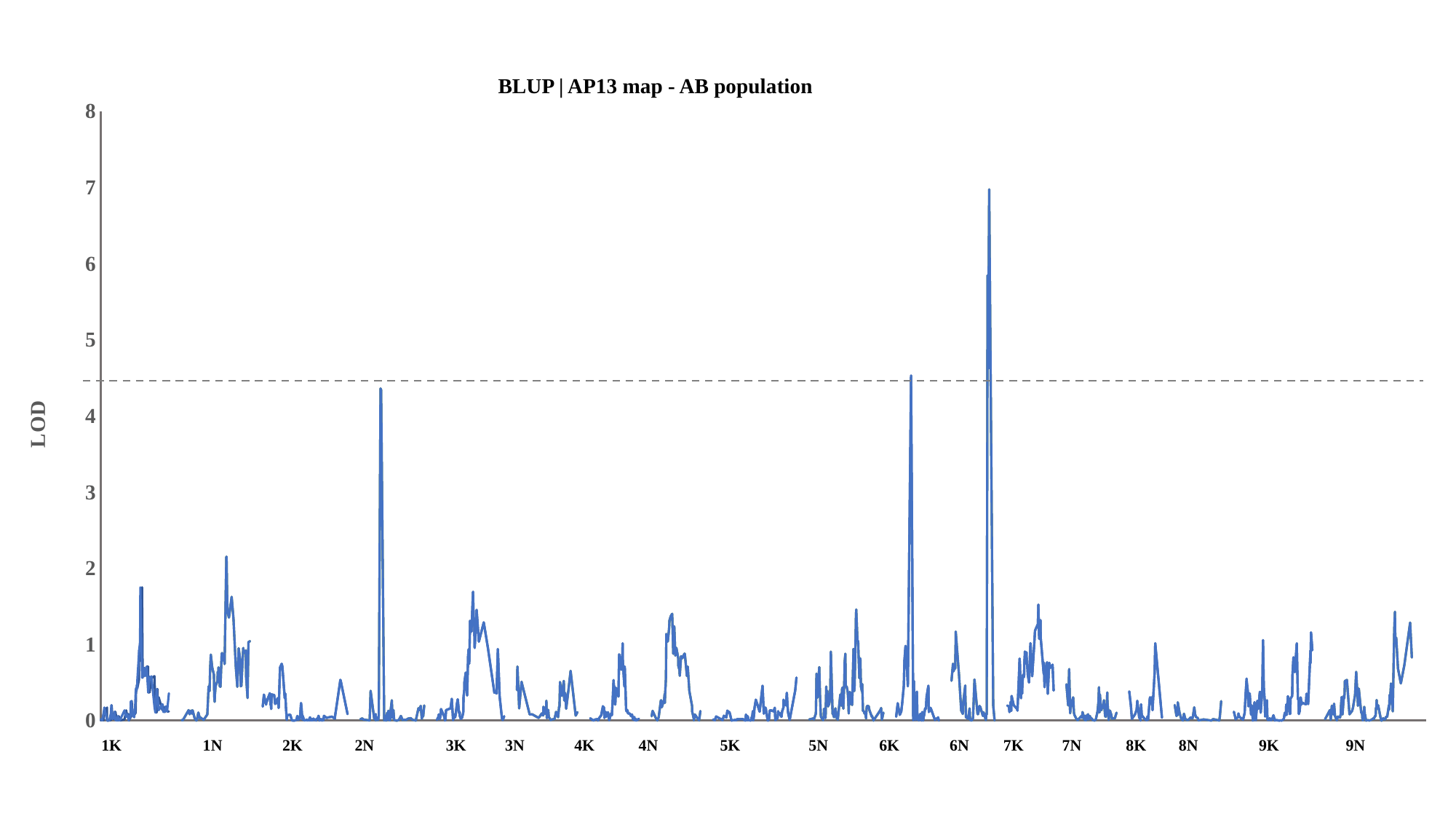

BLUP | AP13 map - AB population
### Chart
| Category | | 1K | 1N | 2K | 2N | 3K | 3N | 4K | 4N | 5K | 5N | 6K | 6N | 7K | 7N | 8K | 8N | 9K | 9N |
|---|---|---|---|---|---|---|---|---|---|---|---|---|---|---|---|---|---|---|---|
1K
1N
2K
2N
3K
3N
4K
4N
5K
5N
6K
6N
7K
7N
8K
8N
9K
9N

## Slide 4
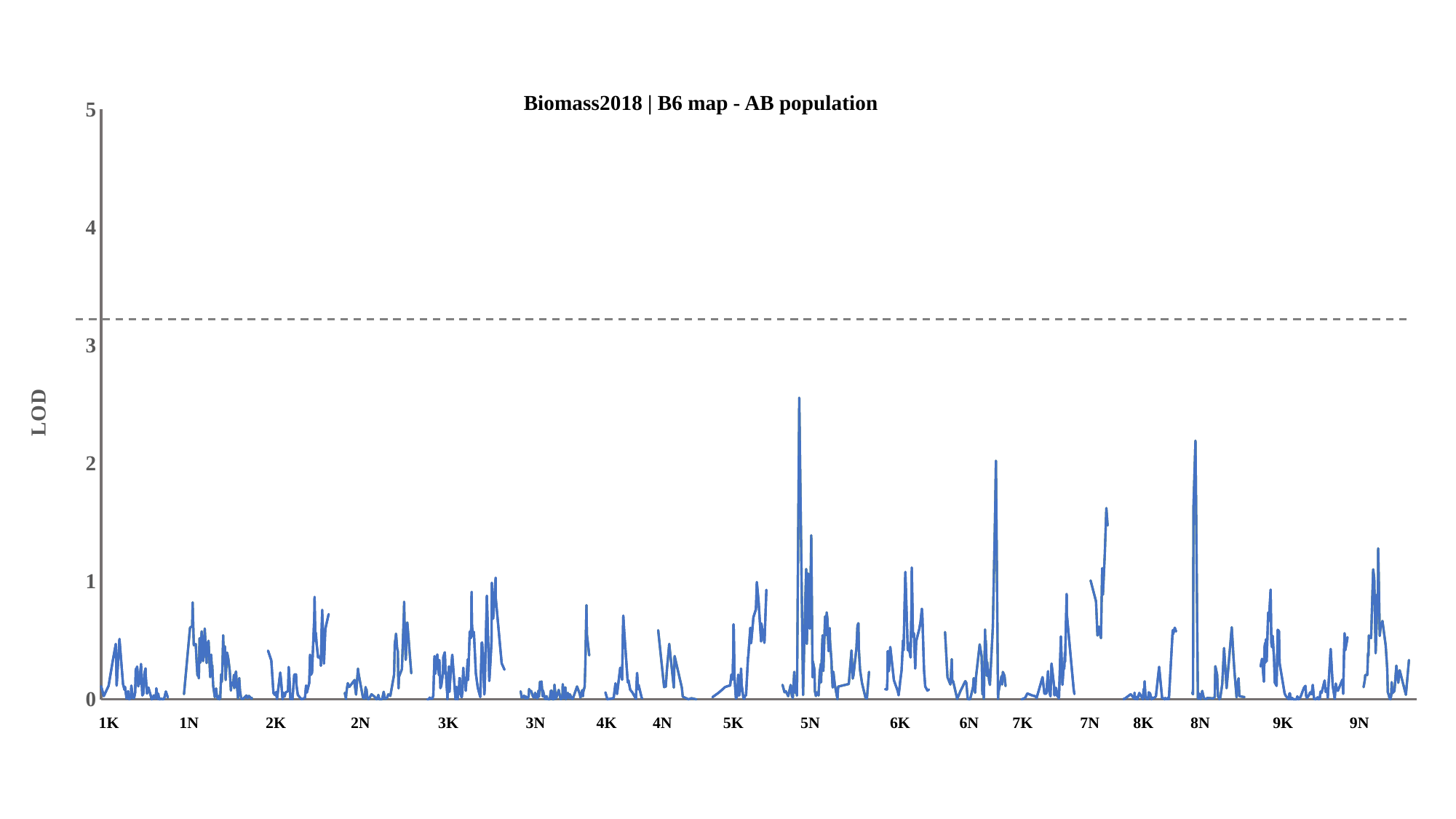

Biomass2018 | B6 map - AB population
### Chart
| Category | Biomass2018-AB | 1K | 1N | 2K | 2N | 3K | 3N | 4K | 4N | 5K | 5N | 6K | 6N | 7K | 7N | 8K | 8N | 9K | 9N |
|---|---|---|---|---|---|---|---|---|---|---|---|---|---|---|---|---|---|---|---|
1K
1N
2K
2N
3K
3N
4K
4N
5K
5N
6K
6N
7K
7N
8K
8N
9K
9N

## Slide 5
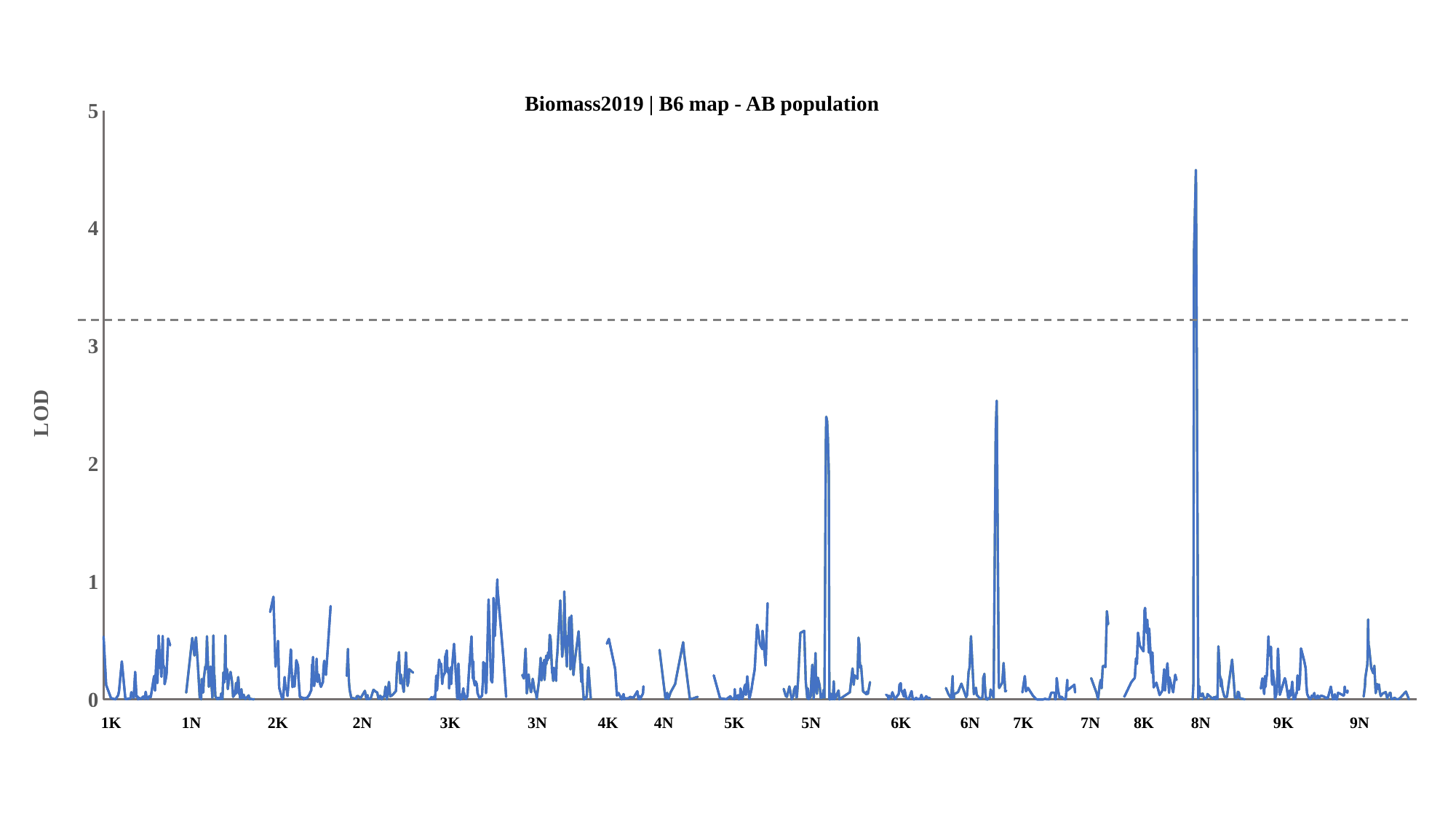

Biomass2019 | B6 map - AB population
### Chart
| Category | Biomass2019-AB | 1K | 1N | 2K | 2N | 3K | 3N | 4K | 4N | 5K | 5N | 6K | 6N | 7K | 7N | 8K | 8N | 9K | 9N |
|---|---|---|---|---|---|---|---|---|---|---|---|---|---|---|---|---|---|---|---|
1K
1N
2K
2N
3K
3N
4K
4N
5K
5N
6K
6N
7K
7N
8K
8N
9K
9N

## Slide 6
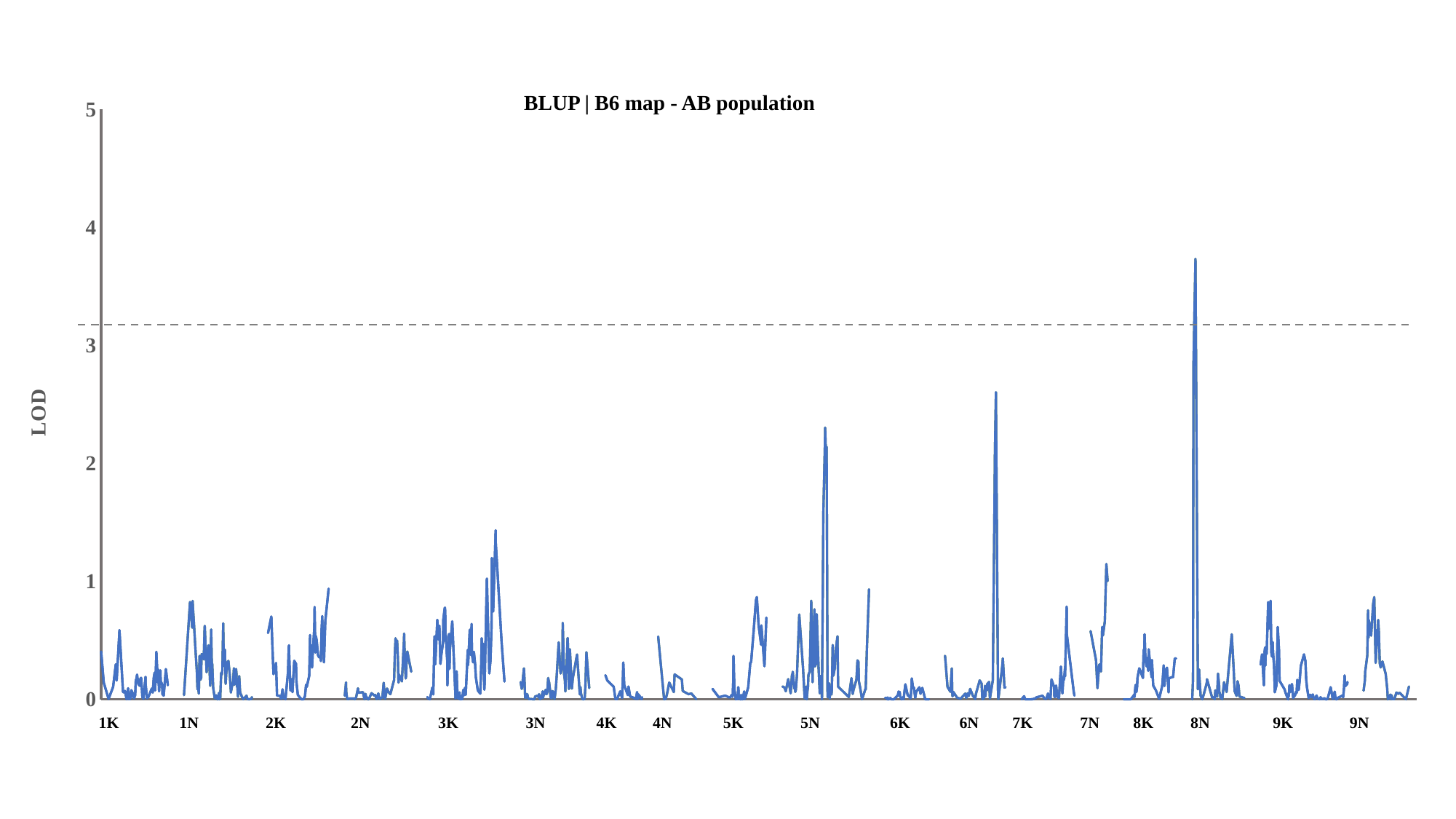

BLUP | B6 map - AB population
### Chart
| Category | BLUP-AB | 1K | 1N | 2K | 2N | 3K | 3N | 4K | 4N | 5K | 5N | 6K | 6N | 7K | 7N | 8K | 8N | 9K | 9N |
|---|---|---|---|---|---|---|---|---|---|---|---|---|---|---|---|---|---|---|---|
1K
1N
2K
2N
3K
3N
4K
4N
5K
5N
6K
6N
7K
7N
8K
8N
9K
9N

## Slide 7
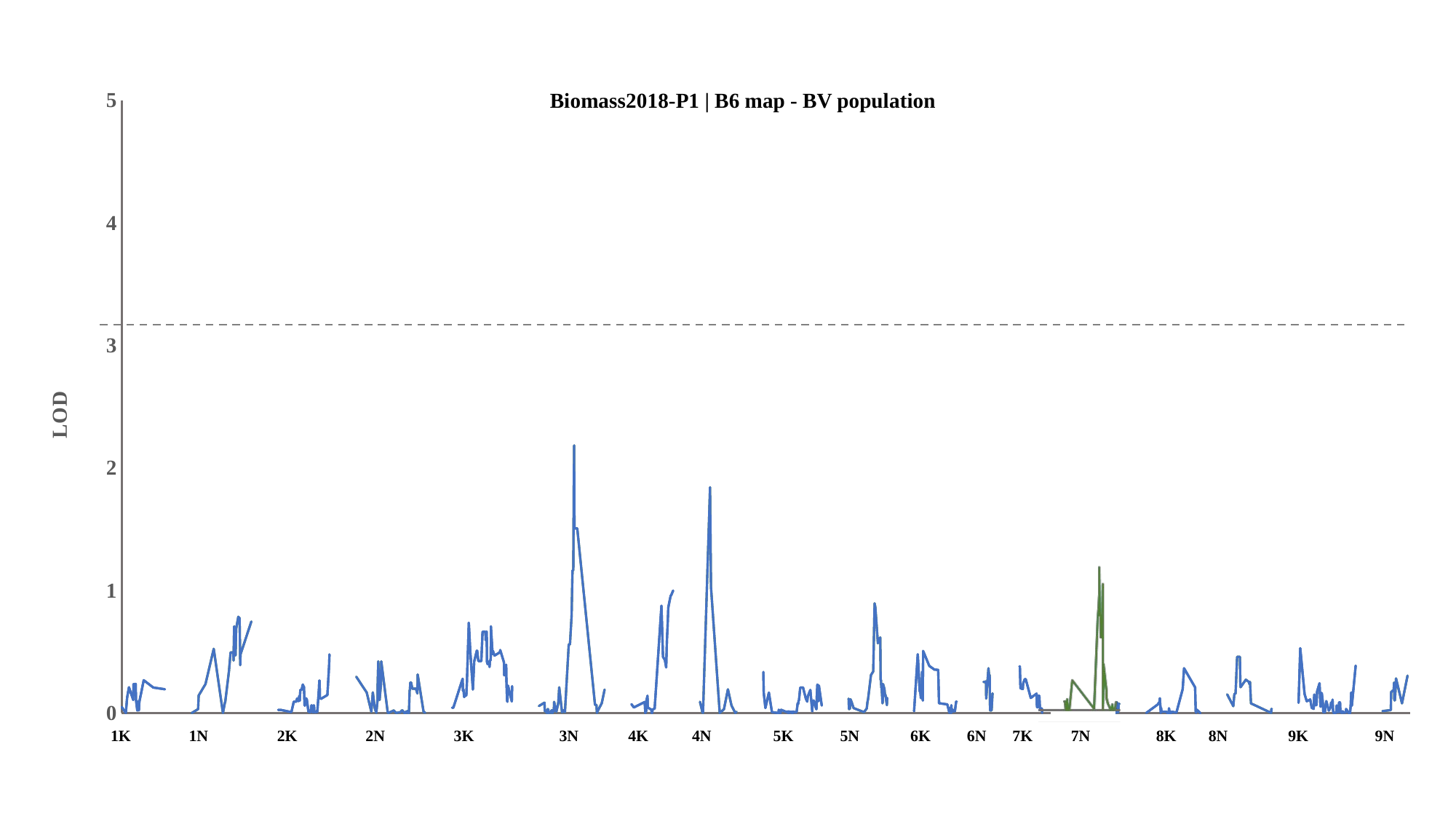

### Chart
| Category | Biomass2018-BVP1 | 1K | 1N | 2K | 2N | 3K | 3N | 4K | 4N | 5K | 5N | 6K | 6N | 7K | 7N | 8K | 8N | 9K | 9N |
|---|---|---|---|---|---|---|---|---|---|---|---|---|---|---|---|---|---|---|---|Biomass2018-P1 | B6 map - BV population
1K
1N
2K
2N
3K
3N
4K
4N
5K
5N
6K
6N
7K
7N
8K
8N
9K
9N

## Slide 8
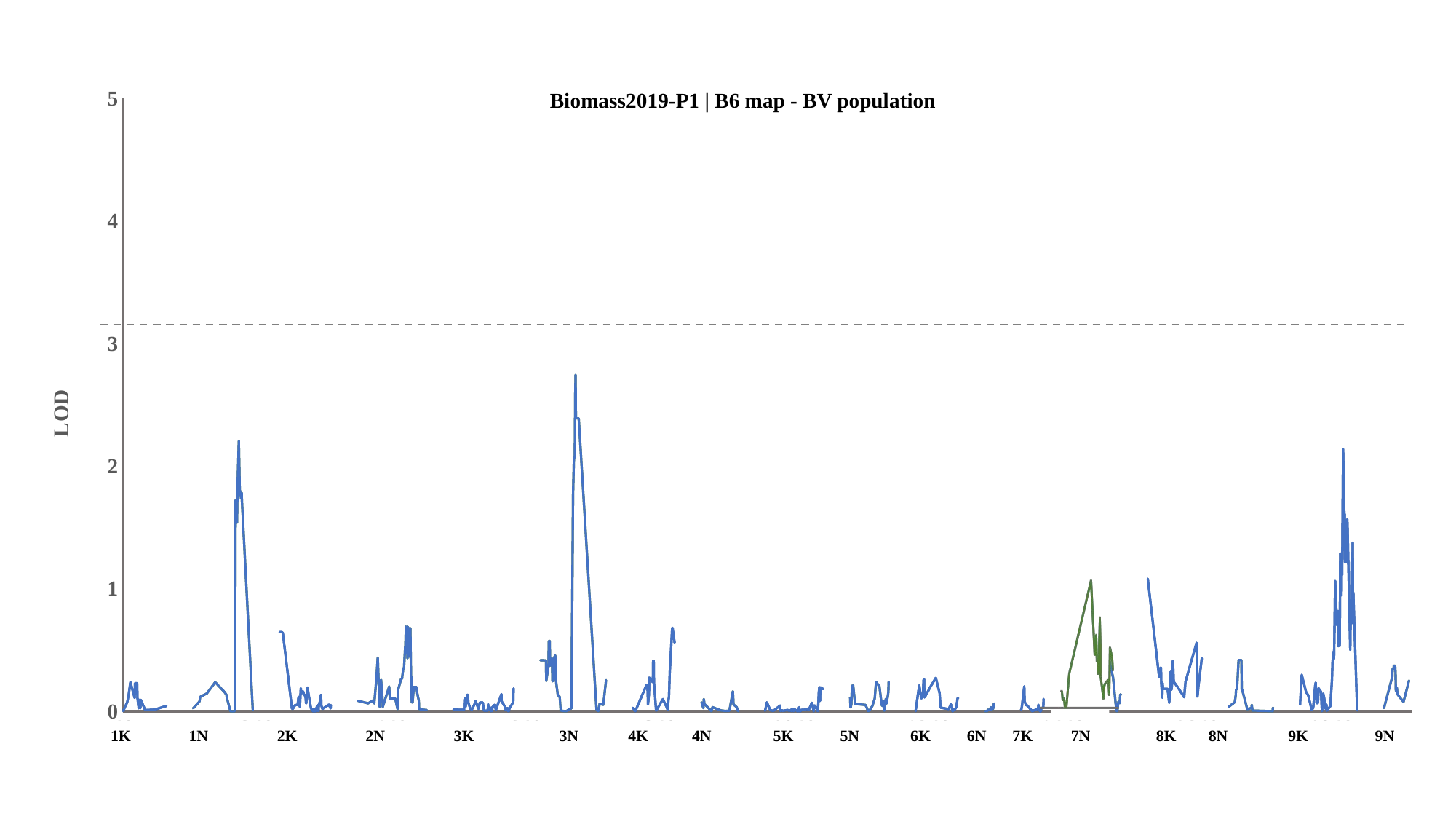

### Chart
| Category | Biomass2019-BVP1 | 1K | 1N | 2K | 2N | 3K | 3N | 4K | 4N | 5K | 5N | 6K | 6N | 7K | 7N | 8K | 8N | 9K | 9N |
|---|---|---|---|---|---|---|---|---|---|---|---|---|---|---|---|---|---|---|---|Biomass2019-P1 | B6 map - BV population
1K
1N
2K
2N
3K
3N
4K
4N
5K
5N
6K
6N
7K
7N
8K
8N
9K
9N

## Slide 9
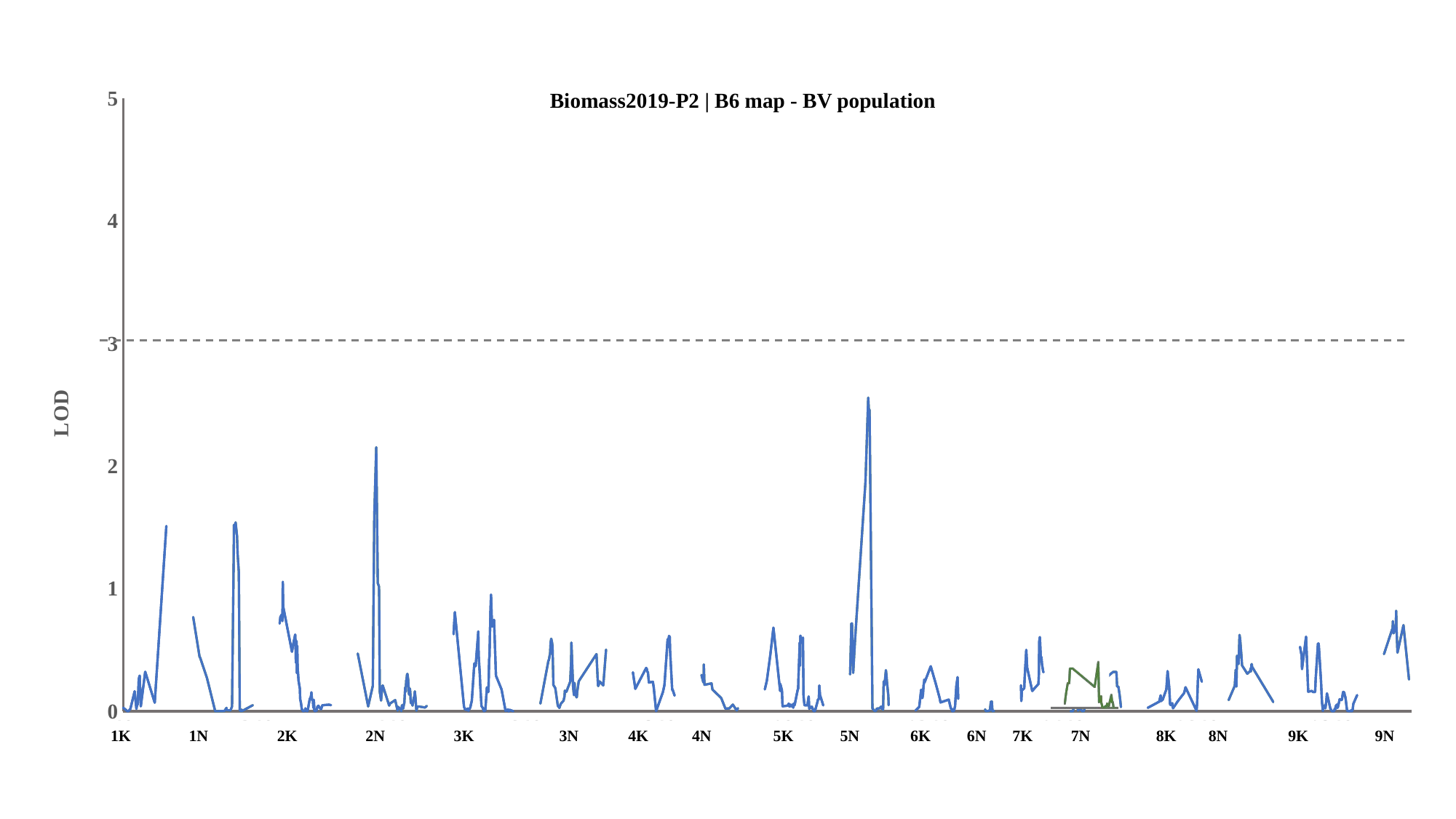

### Chart
| Category | Biomass2019-BVP2 | 1K | 1N | 2K | 2N | 3K | 3N | 4K | 4N | 5K | 5N | 6K | 6N | 7K | 7N | 8K | 8N | 9K | 9N |
|---|---|---|---|---|---|---|---|---|---|---|---|---|---|---|---|---|---|---|---|Biomass2019-P2 | B6 map - BV population
1K
1N
2K
2N
3K
3N
4K
4N
5K
5N
6K
6N
7K
7N
8K
8N
9K
9N

## Slide 10
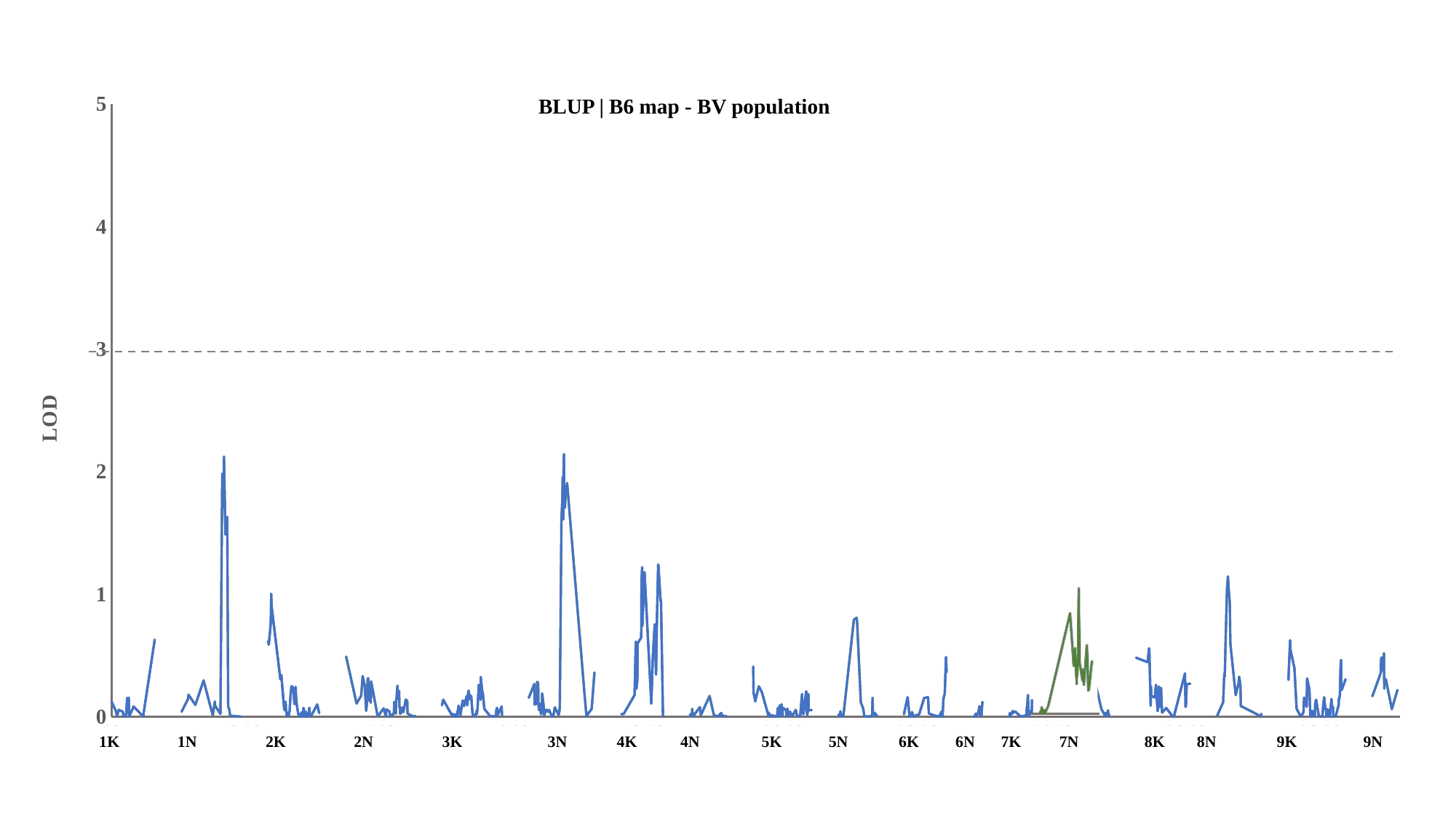

### Chart
| Category | BLUP-BV | 1K | 1N | 2K | 2N | 3K | 3N | 4K | 4N | 5K | 5N | 6K | 6N | 7K | 7N | 8K | 8N | 9K | 9N |
|---|---|---|---|---|---|---|---|---|---|---|---|---|---|---|---|---|---|---|---|BLUP | B6 map - BV population
1K
1N
2K
2N
3K
3N
4K
4N
5K
5N
6K
6N
7K
7N
8K
8N
9K
9N

## Slide 11
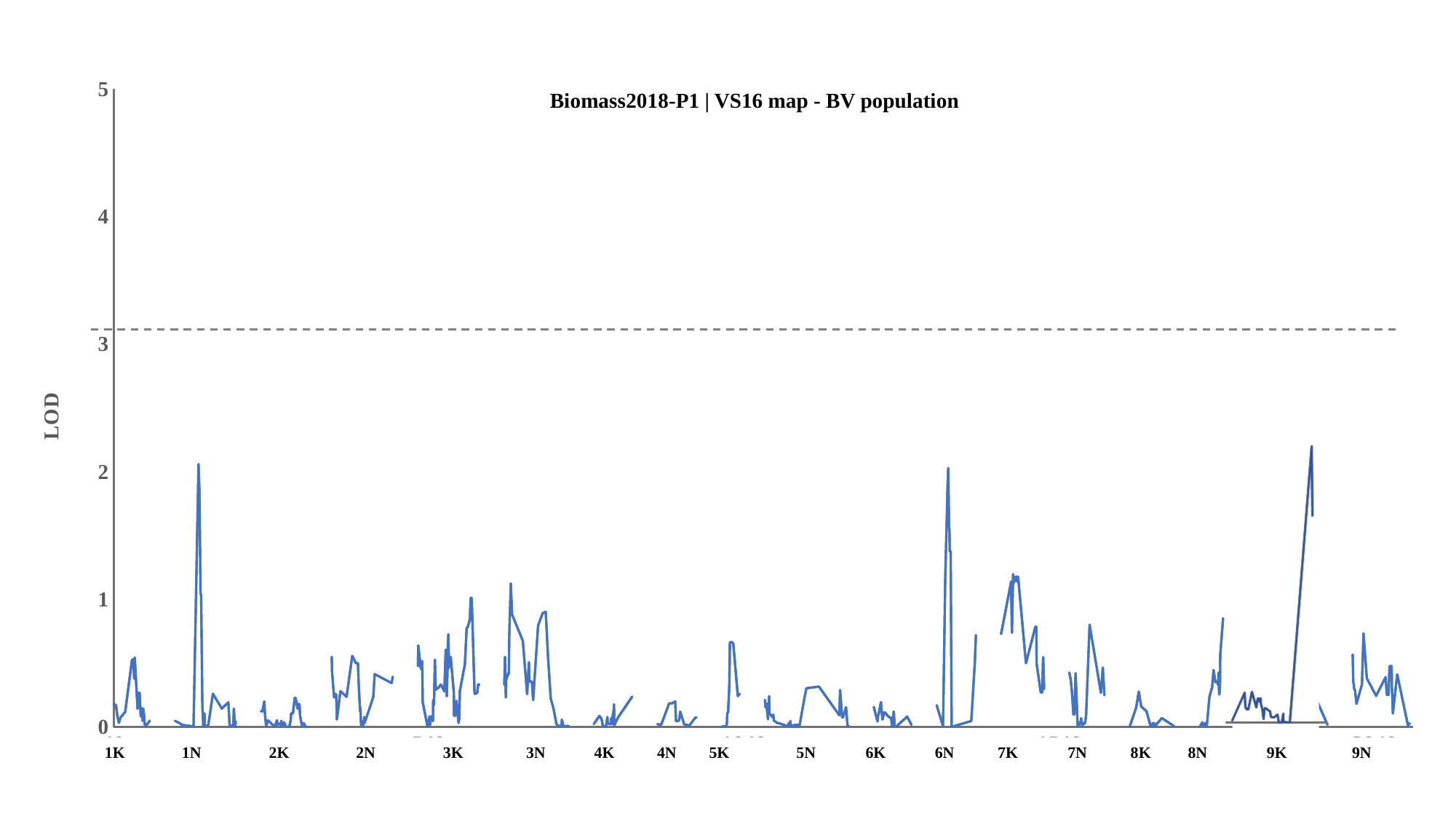

### Chart
| Category | Biomass2018-BVP1 | 1K | 1N | 2K | 2N | 3K | 3N | 4K | 4N | 5K | 5N | 6K | 6N | 7K | 7N | 8K | 8N | 9K | 9N |
|---|---|---|---|---|---|---|---|---|---|---|---|---|---|---|---|---|---|---|---|Biomass2018-P1 | VS16 map - BV population
1K
1N
2K
2N
3K
3N
4K
4N
5K
5N
6K
6N
7K
7N
8K
8N
9K
9N

## Slide 12
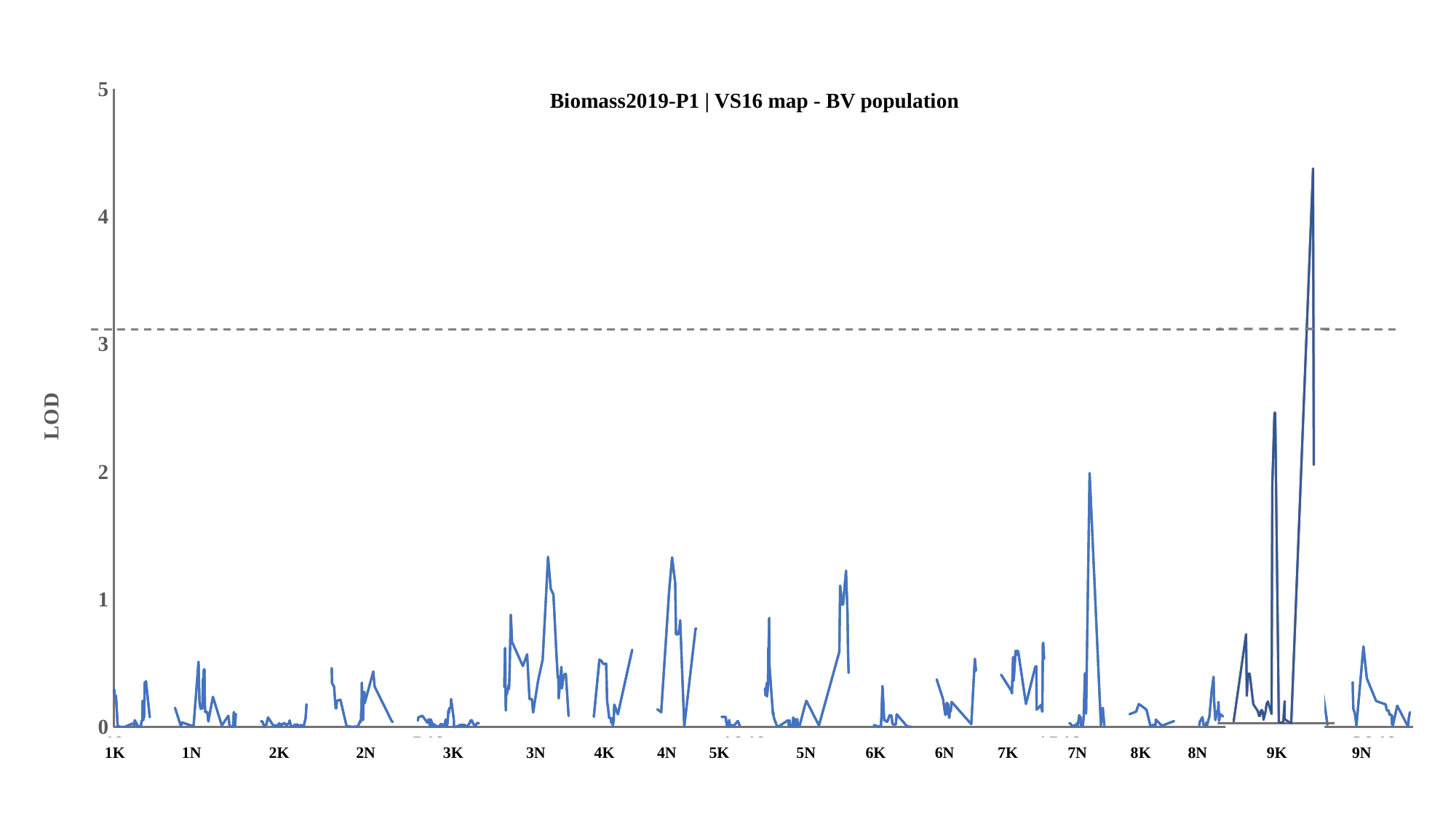

### Chart
| Category | Biomass2019-BVP1 | 1K | 1N | 2K | 2N | 3K | 3N | 4K | 4N | 5K | 5N | 6K | 6N | 7K | 7N | 8K | 8N | 9K | 9N |
|---|---|---|---|---|---|---|---|---|---|---|---|---|---|---|---|---|---|---|---|Biomass2019-P1 | VS16 map - BV population
1K
1N
2K
2N
3K
3N
4K
4N
5K
5N
6K
6N
7K
7N
8K
8N
9K
9N

## Slide 13
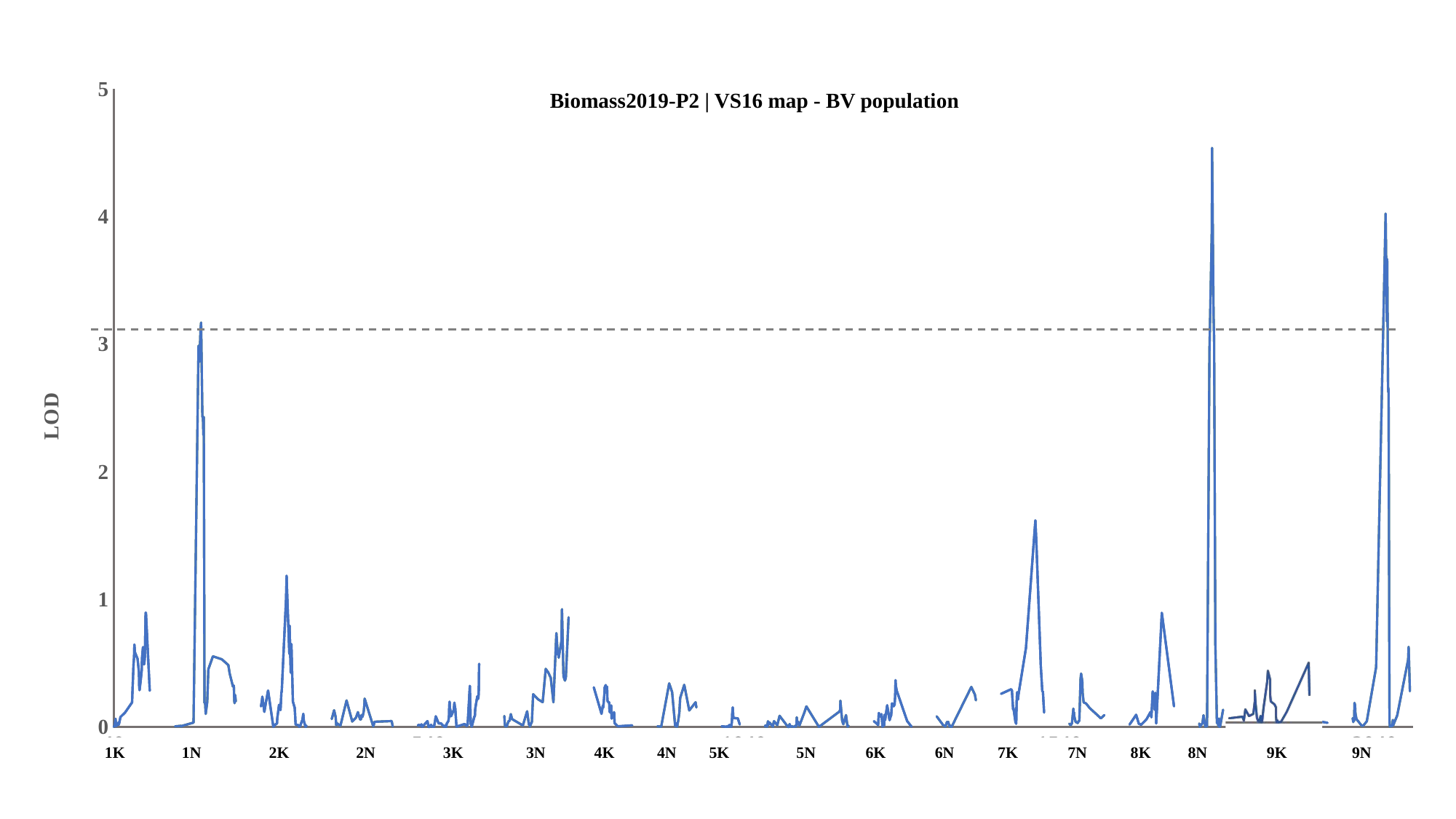

### Chart
| Category | Biomass2019-BVP2 | 1K | 1N | 2K | 2N | 3K | 3N | 4K | 4N | 5K | 5N | 6K | 6N | 7K | 7N | 8K | 8N | 9K | 9N |
|---|---|---|---|---|---|---|---|---|---|---|---|---|---|---|---|---|---|---|---|Biomass2019-P2 | VS16 map - BV population
1K
1N
2K
2N
3K
3N
4K
4N
5K
5N
6K
6N
7K
7N
8K
8N
9K
9N

## Slide 14
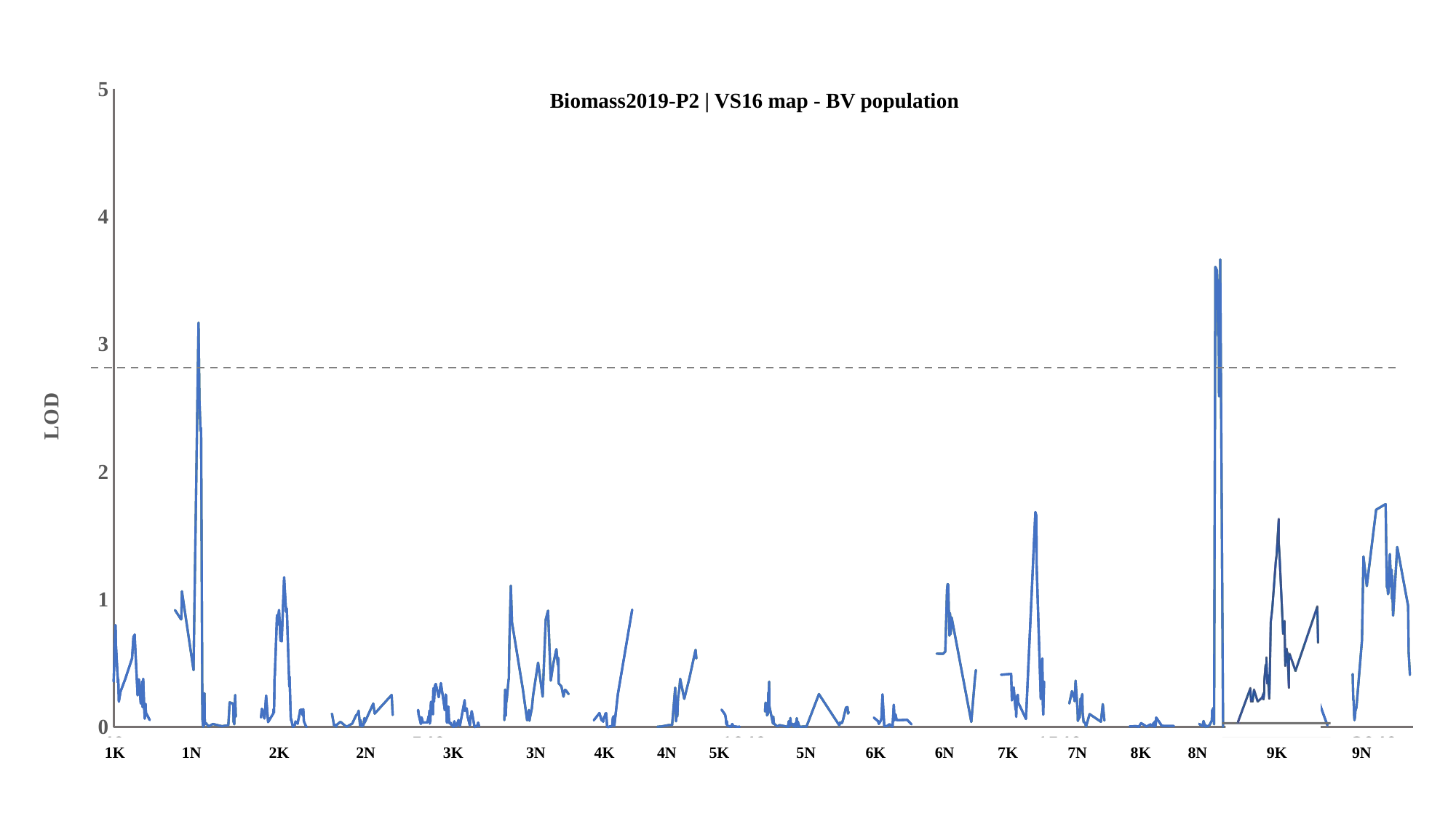

### Chart
| Category | BLUP-BV | 1K | 1N | 2K | 2N | 3K | 3N | 4K | 4N | 5K | 5N | 6K | 6N | 7K | 7N | 8K | 8N | 9K | 9N |
|---|---|---|---|---|---|---|---|---|---|---|---|---|---|---|---|---|---|---|---|Biomass2019-P2 | VS16 map - BV population
1K
1N
2K
2N
3K
3N
4K
4N
5K
5N
6K
6N
7K
7N
8K
8N
9K
9N
